# Model Analysis of the Role of Kinetics, Adsorption Capacity, and Heat and Mass Transfer Effects in Sorption Enhanced Dimethyl Ether Synthesis

**DOI:** 10.1021/acs.iecr.1c00521

**Published:** 2021-03-23

**Authors:** Simone Guffanti, Carlo Giorgio Visconti, Gianpiero Groppi

**Affiliations:** Laboratory of Catalysis and Catalytic Processes, Dipartimento di Energia, Politecnico di Milano, via La Masa 34, Milano 20156, Italy

## Abstract

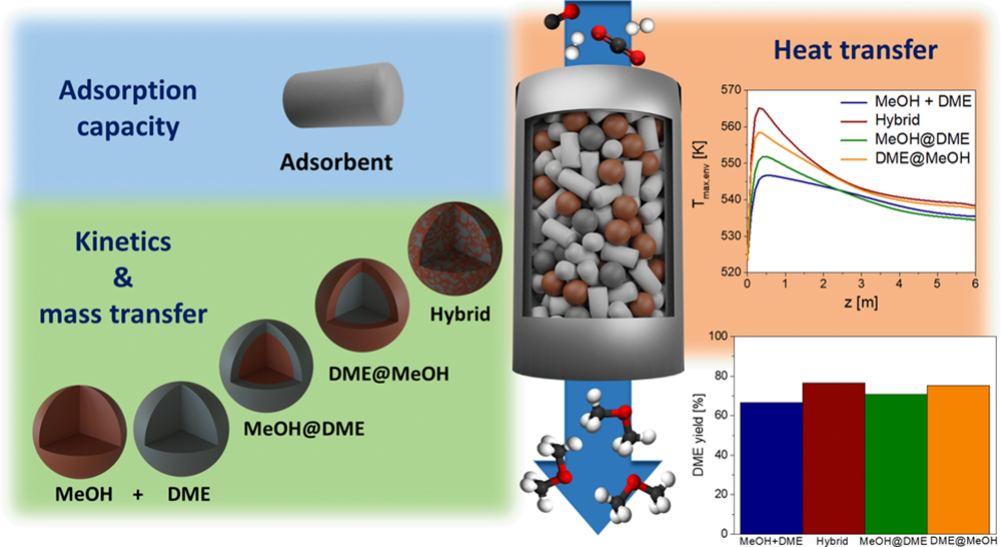

The role of kinetics, adsorption capacity, and heat and mass transfer effects in the sorption enhanced dimethyl ether synthesis (SEDMES) is investigated by means of a 2D+1D model of a single tube of an industrial-scale, externally cooled, multitubular reactor that simulates the reaction/adsorption step of the SEDMES cycle. The effect of the adsorbent/catalyst weight ratio is analyzed, showing that a trade-off between DME productivity and yield originates from the balance of kinetics and adsorption capacity in the reactor tube. The effects of internal diffusion in catalyst particles are shown to have a strong impact on effective reaction rates: significant yield/productivity improvements are obtained when using a mechanical mixture of catalysts with small particle diameters or by rearranging the distribution of the two active phases in hybrid or core@shell pellets. The thermal effects in the reactor, which are increasingly critical upon intensifying the SEDMES process conditions, are also addressed.

## 1 Introduction

Dimethyl ether (DME), widely used as propellant and intermediate for the production of chemicals, is also a strategic alternative synfuel.^1,2^ It can be obtained from synthesis gas produced by reforming/gasification of both fossil fuels (natural gas, coal)^2,3^ and renewable sources such as biomass^4−6^ and urban waste.^7^ Alternatively, DME can be synthesized via CO_2_ hydrogenation, a route of growing interest within the carbon dioxide capture and utilization (CCU)^8−10^ technologies, where green H_2_ obtained from renewable energy is used.

The direct synthesis of DME, requiring an intimate combination of a metallic methanol synthesis catalyst, typically Cu/ZnO/Al_2_O_3_ (CZA),^11^ with an acidic dehydration catalyst (γ-Al_2_O_3_, zeolites, or heteropolyacids)^12−14^ within a single reactor, is widely investigated in the available literature.^13,15−22^ The reactions involved in the process are the following (eqs 1–4):
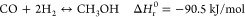
1
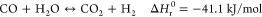
2
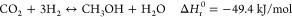
3
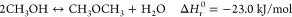
4Compared with the two-stage indirect process, the direct synthesis of DME takes advantage of the thermodynamic synergy of methanol synthesis and dehydration processes:^19,20^ the methanol produced by CO_*x*_ hydrogenation (eqs 1 and 3) is converted via dehydration to DME (eq 4), whereas part of the water produced from reactions 3 and 4 is consumed by water gas shift (WGS) (eq 2).

However, with syngas streams rich in CO_2_, a large production of water occurs, which significantly hinders thermodynamically and kinetically the process, thus lowering the syngas conversion and DME yield per passage^20,21^ and deactivating both the CZA catalyst^19,23,24^ and the γ-Al_2_O_3_.^25^

The in situ reactive steam removal is a possible solution to the issues related to the excess water production in the processes for CO_2_ valorization.^26^ The in situ water removal can be obtained by using either permselective membranes^26−32^ or sorbent materials.^26,33−38^ In the case of direct DME synthesis, high DME selectivity (>95%) can be obtained with both these technologies. The reactive membrane permeation can be advantageous since it does not require a periodic regeneration of the sorbent material, allowing for continuous operation. However, in the case of direct DME synthesis, the reactive adsorption may be preferred since the pressure gradient is the driving force of permeation and low partial pressure of water is required for an effective performance enhancement.^26^

De Falco et al.^31^ reported that a DME yield of 75% can be obtained with a membrane reactor configuration for an optimized case with an operating pressure of 70 bar. Comparable performances are obtained at 30 bar using the reactive steam adsorption.^39−41^ In this process configuration, also known as sorption enhanced DME synthesis (SEDMES), the two catalysts (methanol synthesis and dehydration) used for the direct DME synthesis are mixed with a selective water adsorbent such as LTA zeolite 3A or 4A.^42−45^ The resulting lumped stoichiometries, considering CO and CO_2_ as carbon sources, and the corresponding reaction enthalpies evaluated assuming a water adsorption enthalpy of Δ*H*_ads_ = −46.0 kJ/mol,^44^ are reported in eq 5 and 6, respectively.
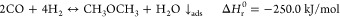
5
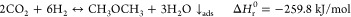
6SEDMES is an intrinsically cyclic process in which the adsorption/reaction step is followed by a regeneration phase, required to remove the water stored in the adsorbent material.^39,46^

This process was investigated both experimentally^24,26,39,46−48^ and theoretically,^46,49,50^ demonstrating the potential of effectively improving the syngas conversion and the DME selectivity with respect to the conventional DME direct synthesis. Van Kampen et al.^46^ simulated the entire SEDMES cycle using a 1D reactor model, analyzing the effects of the process parameters (temperature, pressure, composition, space velocity, adsorbent/catalyst ratio) and the regeneration methods on the cycle performances. The results provide some general guidelines on the choice of operating conditions in SEDMES, displaying the role of reaction kinetics and adsorption capacity on the process performances. By comparing isothermal and adiabatic simulations, temperature control is identified as a critical issue, showing that the negative thermodynamic effect of increasing the temperature drastically reduces the DME yield.

Thermal effects were investigated in detail in a previous paper from our group^50^ by means of a 2D+1D model of a multitubular, externally cooled, industrial-scale SEDMES reactor, which was validated against experimental data. The model simulates the behavior of a SEDMES reactor during the adsorption/reaction step of a pressure swing adsorption (PSA) cycle, well capturing the outlet concentration and temperature profiles measured in the experimental test. The effects of both the CO/CO_2_ ratio in the feed and the tube diameter were analyzed, showing that, thanks to the catalyst dilution with the adsorbent, the maximum thermal stresses are moderate with respect to the conventional DME synthesis,^17,22,51^ despite the additional heat released by water adsorption.

In this work, the modeling analysis of SEDMES full-scale reactors is extended by focusing on the role of the solid materials in the reactor: the adsorbent (zeolite 3A) and the catalysts (CZA and γ-Al_2_O_3_). The two main competing factors determining the SEDMES process performances are indeed the capacity of the system to remove water by adsorption and the rate of DME production. Once the operating conditions as temperature and pressure are fixed, the adsorption capacity and reaction kinetics can be managed by acting on the regeneration methods, on the time-design of the regeneration cycle and on the adsorbent/catalyst ratio.^39,46^ The effect of this latter parameter is addressed in the present analysis, considering its kinetic and thermodynamic consequences and their impact on the thermal behavior of the reactor. In addition, the effect of the active phase distribution at the pellet scale is investigated to address the impact of the intraparticle diffusion limitations in catalyst pellets, which were shown to markedly affect kinetics, and consequently the reactor performances in direct DME synthesis.^51−55^

## 2 Methods

### 2.1 SEDMES Reactor Model

A 2D heterogeneous dynamic model of a SEDMES reactor, originally developed and validated for a mechanical mixture of methanol synthesis and dehydration catalysts and the adsorbent,^50^ is adopted in this work. The model describes a single tube of an externally cooled multi tubular fixed bed reactor packed with pellets of LTA zeolite 3A adsorbent and two k-catalyst phases: CZA methanol (MeOH) synthesis catalyst and γ-Al_2_O_3_ methanol dehydration to DME catalyst (DME). The model includes 2D total mass and energy balances for the gas phase and 2D *i*-species (*i* = CO, CO_2_, H_2_, H_2_O, CH_3_OH, DME, N_2_) mass balances for the gas phase as well as for catalyst and adsorbent solid phases (Tables 1 and 2). Pressure drops are neglected because of the low gas velocities considered (<0.03 m/s). The dynamic reactor model is coupled with pseudostationary *i*-species 1D mass balances within the catalyst pellets (Table 3), which account for the intraparticle diffusion limitations.

**Table 1:**
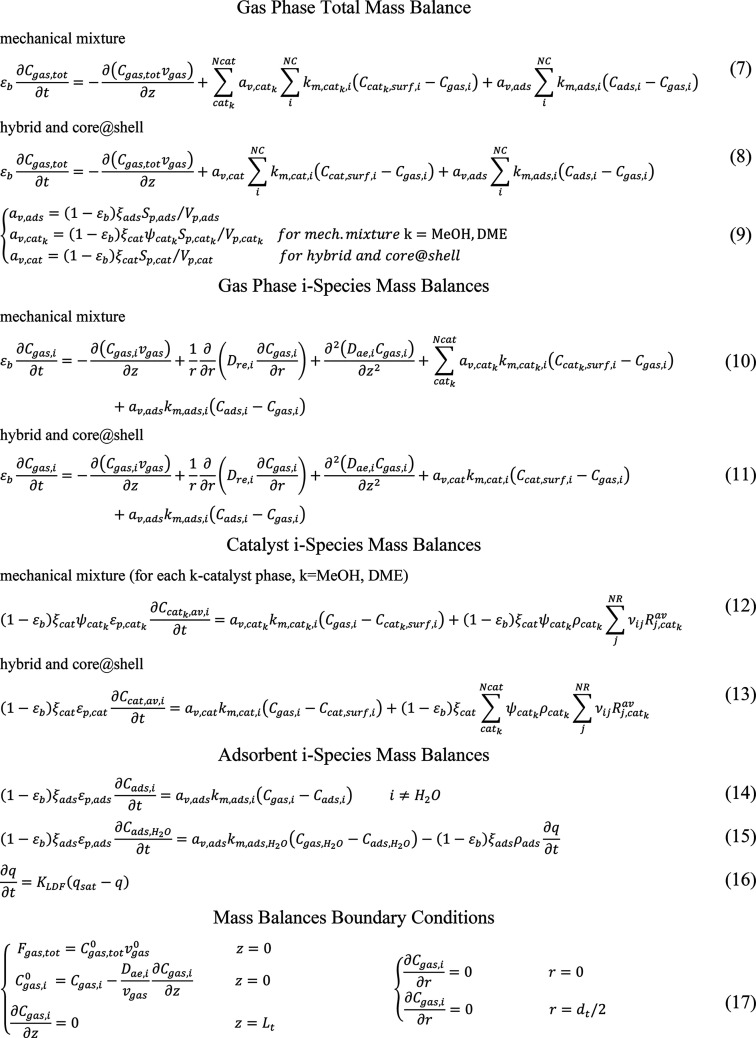
2D Reactor Model Mass Balance Equations

**Table 2:**
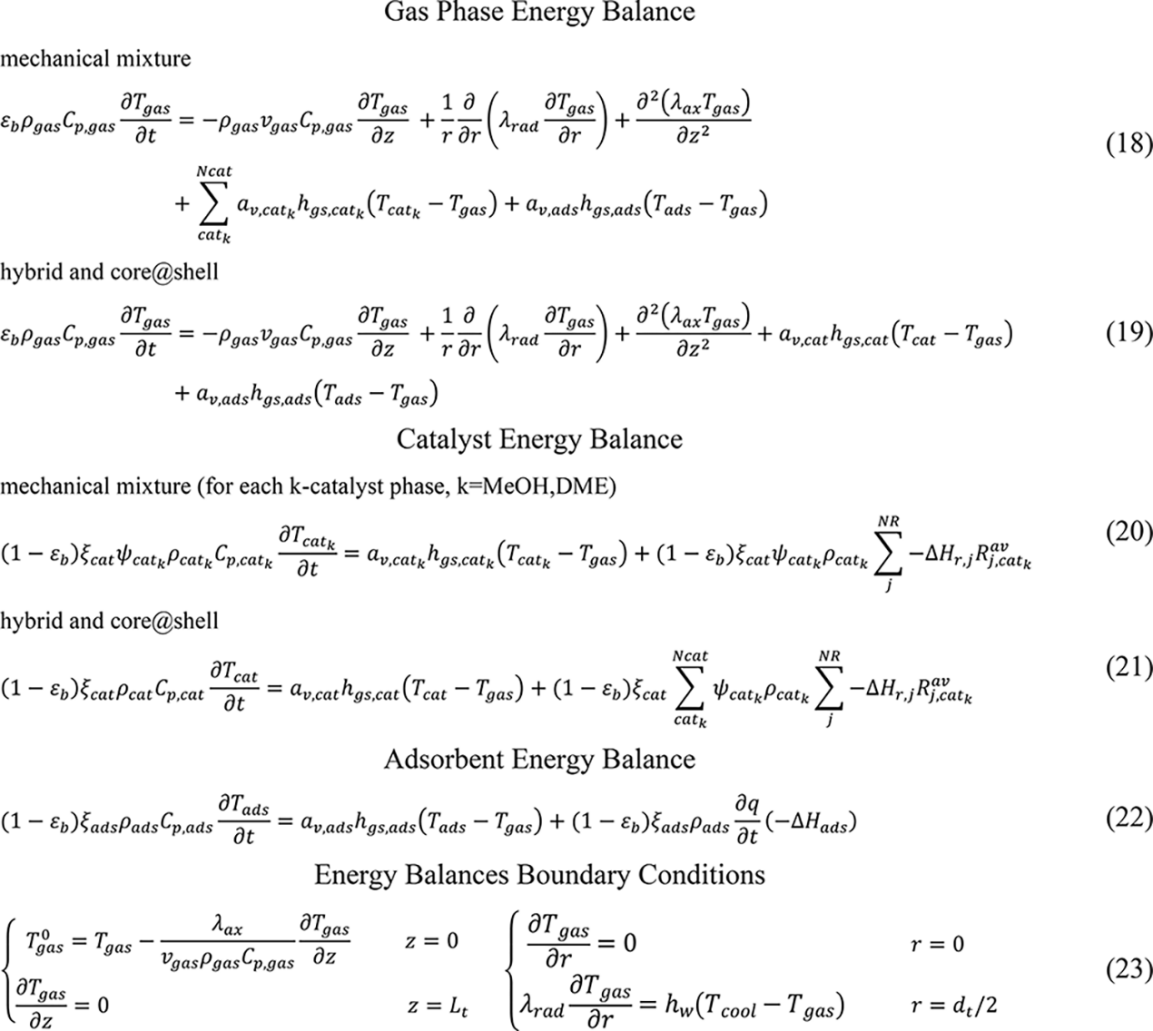
2D Reactor Model Energy Balance Equations

**Table 3:** 1D Pellet Model Mass Balances

Pellet *i*-Species Mass Balances
mechanical mixture (for each *k*-catalyst phase, *k* = MeOH, DME)
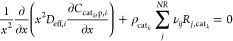 24
hybrid and core@shell
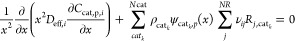 25
for hybrid
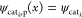 26a
for MeOH@DME
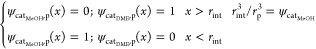 26b
for DME@MeOH
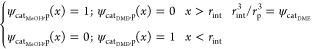 26c
Average Reaction Rates and *i*-Species Catalyst Concentration
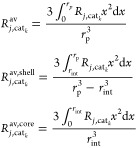 27
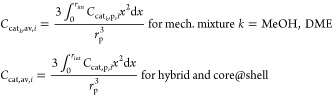 28
Pellet Mass Balance Boundary Conditions
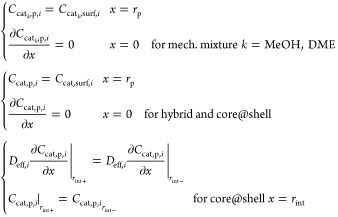 29

**Figure 1:**
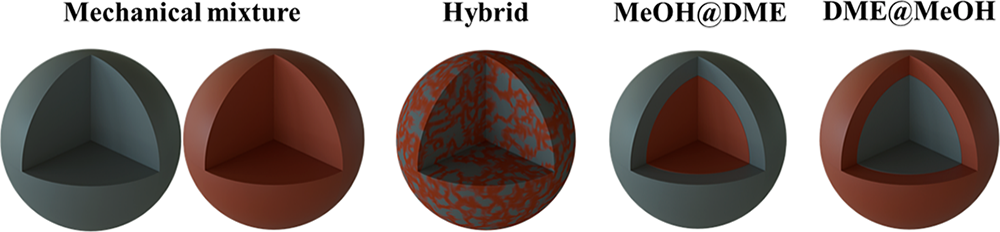
Catalyst pellet configurations sketch. Brown, CZA (MeOH) catalyst; gray, γ-Al_2_O_3_ (DME) catalyst.

The model is herein extended in order to account for different catalyst pellet configurations (Figure 1): mechanical mixture, hybrid, MeOH@DME, and DME@MeOH. In the mechanical mixture configuration, CZA pellets (brown in Figure 1) for methanol synthesis are mixed with γ-Al_2_O_3_ pellets (gray in Figure 1) for methanol dehydration. The hybrid pellet is a configuration with the catalytic materials interdispersed inside the same particle, creating a homogeneous active phase distribution.^56^ The core@shell configurations instead, consist in the layering of the methanol synthesis and dehydration active phases in a single pellet.^51,54,55,57−66^ In the case of MeOH@DME configuration, the CZA catalyst core (brown) is surrounded by a shell of γ-Al_2_O_3_, (gray), whereas in the DME@MeOH, the core and the shell formulations are inverted.

The main changes herein introduced with respect to the original model are related to the presence of only one type of catalyst pellet in hybrid and core@shell (two types of catalyst pellets are present in the mechanical mixture) and in the different distribution of MeOH and DME active phases within the particles in these configurations, accounted for by the parameter ψ_cat_*k*_,p_(*x*) (see Table 3). More details on this aspect are reported in a previously published paper,^51^ describing a similar analysis for the conventional direct DME synthesis.

### 2.2 Transport Correlations, Physical Properties, Reaction Kinetic Scheme, and Adsorption Isotherm

Diffusivities, as well as mass and heat transport coefficients, are calculated using literature correlations (Supporting Information S1), whereas physical and chemical properties (molecular weight, specific heat, density, viscosity, and thermal conductivity) of the reacting mixture are calculated using the *gPROMS Multiflash* 4.3 utility tool. The solid phases physical properties, taken from,^43,46,67−69^ are reported in Table 4, with densities depending on the solid type and conductivity and specific heat capacity fixed to typical values for porous ceramics.

**Table 4:** Physical Properties of Solid Phases

parameter	value	unit
*ρ*_MeOH_	1712	kg/m^3^
*ρ*_DME_	1285	kg/m^3^
*ρ*_ads_	1200	kg/m^3^
*C*_p,s_	960	J/(kg K)
*λ*_s_	0.22	W/(m K)
Δ**H*_*ads*_*	–45.95	kJ/mol_H_2_O_

The reactions considered in the kinetic scheme are (1) methanol synthesis from CO, (2) reverse WGS (at SEDMES condition the shift reaction proceeds reversely due to the low fraction water), (3) methanol synthesis from CO_2_, and (4) methanol dehydration to DME. The rate expressions associated with the CZA methanol synthesis catalyst (eqs 30–32) are taken from Graaf et al.,^70^ whereas the rate law for methanol dehydration to DME (33) is taken from Ng et al.^71^
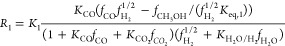
30
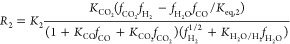
31
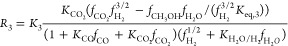
32
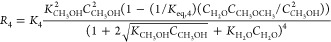
33Kinetic parameters taken from the literature are reported in Supporting Information S2 together with the equilibrium constants (*K*_eq,1_–*K*_eq,4_).^72,73^ According to the results of the model validation carried out in a previous work,^50^ a multiplicative factor of 5 is applied to the CO_*x*_ hydrogenation reaction rates and a factor of 7.5 to the reverse WGS calculated with eqs 30 and 32 and eq 31, respectively. This is reasonable, as the CZA catalyst are found to have higher activity in standard methanol synthesis conditions,^67^ and the catalyst is even more active in SEDMES operating conditions because of the very low concentration of water secured by in situ adsorption.^47^

The catalyst pellets are homogeneously mixed with the LTA zeolite 3A particles, which selectively adsorb water. The adsorption kinetic is calculated considering the linear driving force approximation (see eq 16 in Table 1). A Langmuir–Freundlich isotherm model (eq 34) is used in the simulations, using the parameters proposed by Gabruś et al.^44^
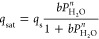
34The adsorption isotherm parameters are reported in Supporting Information S3.

### 2.3 Numerical Solution Scheme

The SEDMES reactor model equations, together with physical and transport correlations, rate expressions, and adsorption isotherm equations, are implemented in *gPROMS* software for the numerical solution. A standard differential-algebraic equation system solver based on an implicit backward differentiation formula (BDF) with variable time step and variable order is used for time integration. The integration time step of BDF is automatically adjusted by the *gPROMS* algorithm in accordance with a maximum local error criterion, whereas the integration order changes between one (corresponding to an implicit Euler) to four. The first-order backward finite difference method (BFDM) is used for the discretization of the axial reactor coordinate, whereas third-order orthogonal collocations on the finite elements method (OCFEM) are used for the radial and the pellet coordinates. An equi-spaced grid of 60 discretization points is used along the reactor axial coordinate, with two finite elements for the reactor radial coordinate. The pellet coordinate is discretized using two finite elements collocations in mechanical mixture and hybrid pellet cases, whereas two elements are used for the core and two for the shell in the core@shell cases. The adequacy of the number of discretization points and finite elements is checked by a convergence analysis.

### 2.4 Simulation Input Variables

The geometrical and operating conditions input parameters used are reported in Table 5. An industrial scale multitubular fixed bed reactor externally cooled by boiling water with 6 m length and 38 mm diameter tubes is considered. The reactor operates at 25 bar with 523 K as the gas inlet and coolant temperature and a gas hourly space velocity (GHSV), referred to the total catalyst volume, of 805 h^–1^. The inlet gas composition (Table 6) has a module *M* = (H_2_ – CO_2_)/(CO + CO_2_) = 2, which is the thermodynamic optimum for the SEDMES process,^26^ and a ratio CO/CO_2_ = 1. A CZA/γ-Al_2_O_3_ ratio of 1/1 w/w is assumed as a typical value in SEDMES process.^24^

**Table 5:** Geometrical Parameters and Operating Conditions of the Reactor Tube

variable	value	unit
*L*_t_	6	m
*d*_*t*_	3.8 × 10^–2^	m
*ρ*_bed_	800	kg/m^3^
MeOH/DME catalyst ratio	1/1	kg/kg
*T*_g_^0^	523	K
*T*_cool_	523	K
*P*^0^	25	bar
GHSV_cat_	805	h^–1^

**Table 6:** Inlet Feed Composition

species	molar %
CO	13.4
CO_2_	13.4
H_2_	66.9
N_2_	6.3

A reaction/adsorption time of *t* = 3600 s is taken as a reference value.^50^ At time zero, the reactor is filled by a purge gas containing 98.5% N_2_ and 1.5% H_2_ at 25 bar and 523 K uniform temperature. The water load profile at time zero *q*_0_ is evaluated by simulating 5400 s of purging with an inert N_2_ stream at 1.5 bar, a specific molar flow rate of 18.6 mol/(m^2^ s), and wall temperature of 523 K, for a reference case, corresponding to the mechanical mixture with a 4/1 w/w adsorbent/catalyst ratio.^50^

## 3 Results and Discussion

The reactor performances are assessed by considering as indicators: (i) the time evolution of the specific outlet DME flow rate and the correspondent DME normalized flow rate F*_C→DME_ = 2*F*_DME,out_/(*F*_CO,in_ + *F*_CO_2_,in_); (ii) the DME carbon yield and productivity, and (iii) the CO_2_ outlet molar fraction and conversion. It is worth noticing that, because the regenerations steps are not simulated, only approximated DME carbon yield (*Y*_DME_), DME productivity (Prod_DME_), and CO_*x*_/CO_2_ conversion (Conv_CO*x*_) can be calculated according to eqs 35–37, assuming that the reactions are kinetically frozen (rate = 0) at the end of the reaction/adsorption step and that all products present in the reactor tube are recovered during the blowdown.
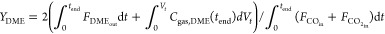
35
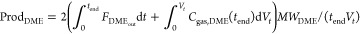
36
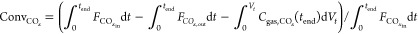
37The other key parameter considered in the analysis is the envelope of maximum temperatures, which is the profile of the highest temperature reached in each axial position during the entire adsorption/reaction step. This parameter is used as indicator of the thermal stresses that must be controlled in order to prevent CZA catalyst deactivation.

### 3.1 Effect of Adsorbent/Catalyst Ratio

The adsorbent/catalyst ratio is a crucial parameter in SEDMES, which governs the trade-off between the adsorption capacity of the system and the kinetics of DME production. A lack of adsorbent reduces the water removal capacity, a more frequent regeneration is required, thus increasing the operational and equipment costs. On the other hand, a lack of catalyst kinetically limits the process, decreasing the productivity per unit volume of the reactor.^46^

The effect of adsorbent/catalyst weight ratio is investigated in the range between 2/1 and 16/1 w/w considering a mechanical mixture configuration with catalyst pellet diameter *d*_p_ = 3 mm and adsorbent pellet diameter *d*_pe_ = 3.2 mm. Simulations are performed at constant GHSV (805 h^–1^) referred to the catalyst volume: the corresponding specific molar flow rate (*F*_tot_) based on the tube cross section, that changes depending on the adsorbent to catalyst ratio, is reported in Table 7.

**Table 7:** Specific Molar Flow Rate As a Function of the Adsorbent/Catalyst Weight Ratio

ads/cat ratio(kg/kg)	*F*_tot_ (mol/m^2^/s)
2/1	17.7
4/1	10.4
8/1	5.7
16/1	3.0

**Figure 2:**
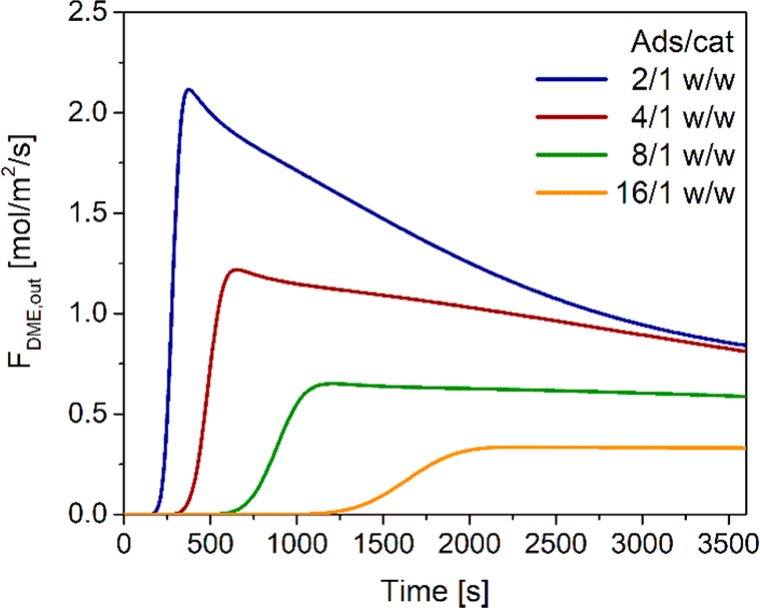
Time evolution of outlet DME specific flow rate with different adsorbent/catalyst weight ratios.

**Figure 3:**
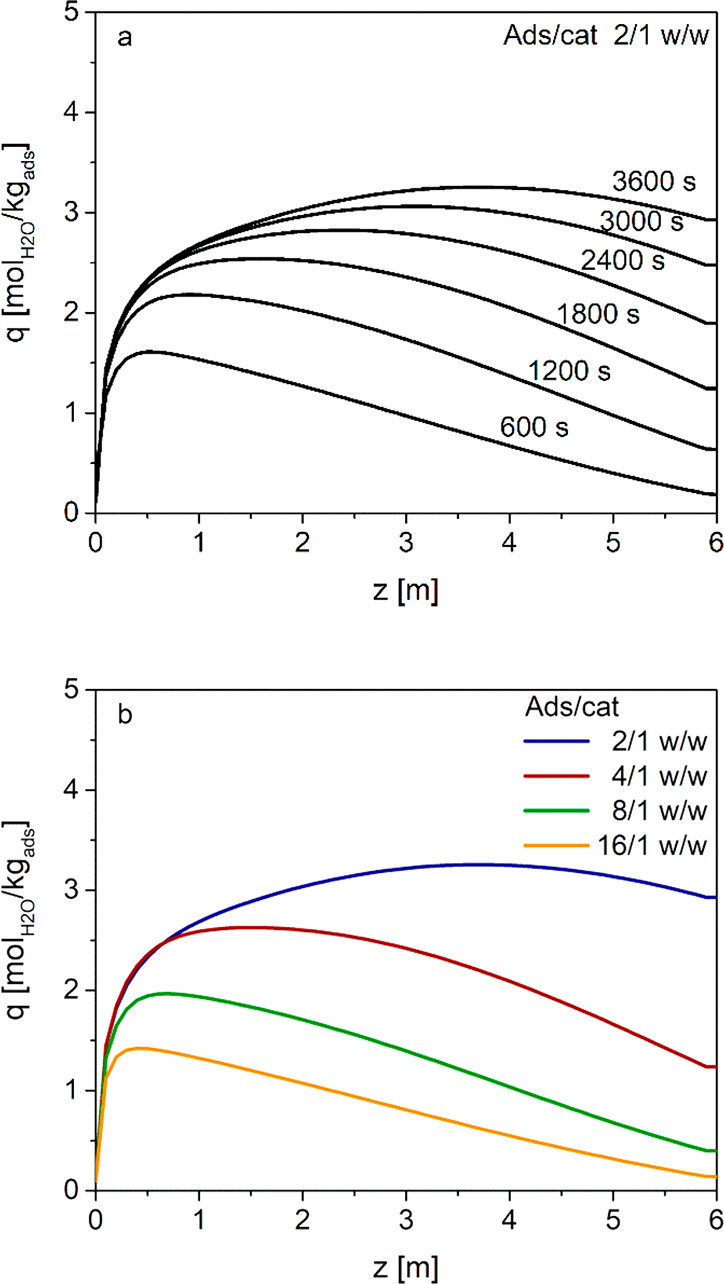
Axial profile of the cross-sectional average adsorbent water load (a) as a function of time with an adsorbent/catalyst ratio 2/1 w/w and (b) at a time of 3600 s for different adsorbent/catalyst weight ratios.

The time evolution of outlet DME specific flow rate is reported in Figure 2. The DME flow rate is null in the first part of the adsorption/reaction step, since at time zero the reactor is full of inert N_2_ and the reactants/products wave takes time before breakthrough. After the breakthrough, the DME flow rate rapidly raises to a maximum and then progressively decreases because of the growing H_2_O holdup of the adsorbent (Figure 3a).

Coherently with the increasing specific flow rate used in the simulations, the peak of the outlet DME flow rate breaks through earlier and grows higher on decreasing the adsorbent to catalyst ratio. On the other hand, the decrease in DME flow rate after the peak becomes steeper on decreasing the adsorbent/catalyst ratio because of the lower adsorption capacity. This results in a higher water hold-up (Figure 3b): with a 2/1 w/w ratio, at 3600 s, the outlet DME flow rate decreases to values similar to those obtained with the 4/1 w/w ratio, whereas with the 16/1 w/w ratio, after the breakthrough, the outlet DME flow rate keeps almost constant with time until the end of adsorption/reaction step.

**Figure 4:**
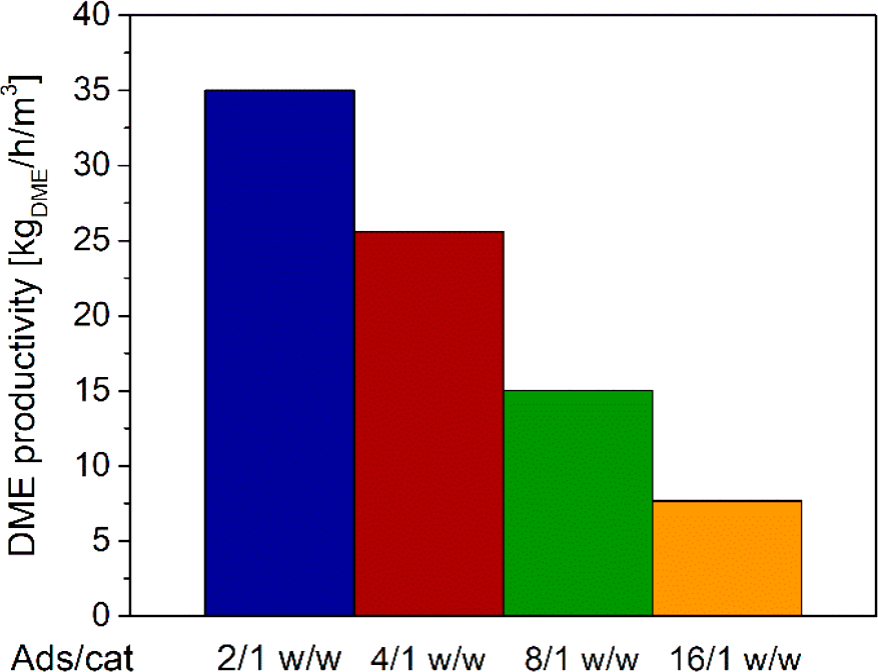
DME productivity with different adsorbent/catalyst weight ratios.

Results in Figure 2 indicate that the overall amount of DME produced along the cycle increases on decreasing the adsorbent catalyst ratio. The DME productivity values, computed with eq 36, which also accounts for the DME recovered in the blow down step, clearly show the extent of productivity increase on decreasing the adsorbent/catalyst ratio (Figure 4).

**Figure 5:**
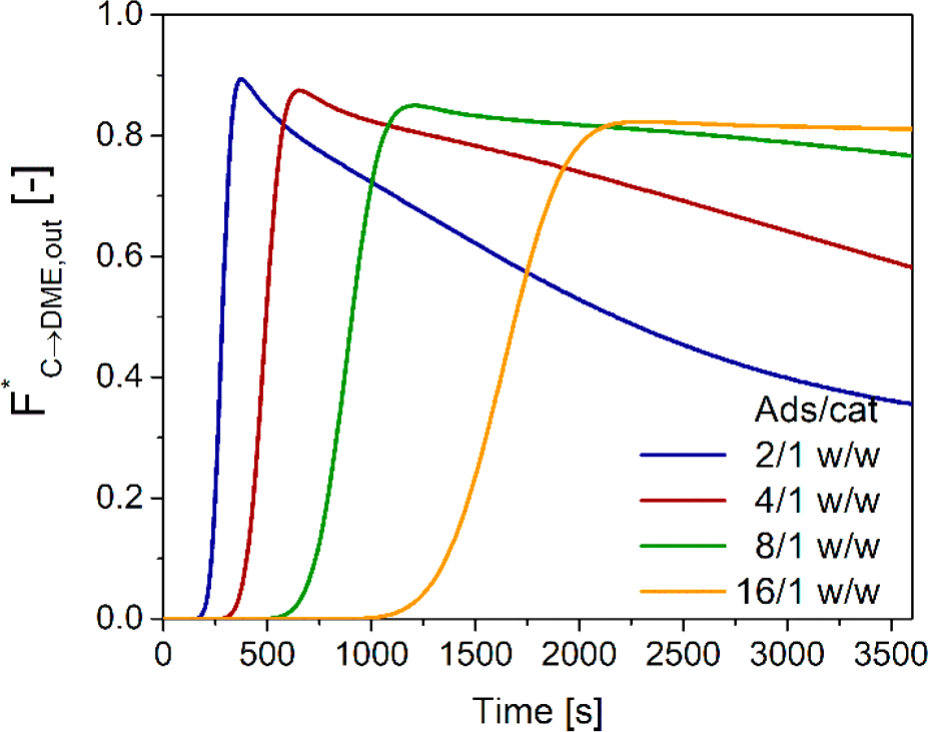
Time evolution of outlet DME flow rate normalized with respect to inlet carbon with different adsorbent/catalyst weight ratios.

A different picture is obtained when normalizing the DME outlet flow rate to the amount of feed carbon (Figure 5). In this case, the maximum, occurring soon after the breakthrough, is almost independent of the adsorbent to catalyst ratio, consistently with a kinetic control at constant GHSV. The role of the adsorption capacity is evidenced by the steeper decay of the DME normalized flow rate on decreasing the adsorbent/catalyst ratio. With 2/1 w/w, the plateau of concentration, corresponding to the conventional DME direct synthesis equilibrium (without sorption enhancement), is almost reached at the end of the reaction/adsorption step because of complete saturation of the water adsorption capacity. Conversely, almost no decay is observed in the case of 16/1 w/w, thanks to the oversized adsorbent load.

**Figure 6:**
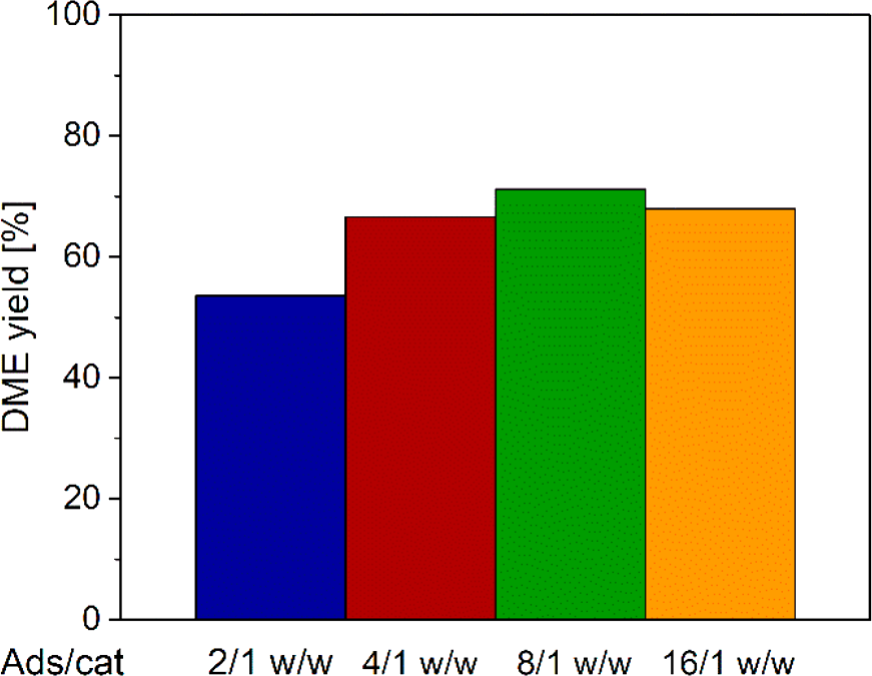
DME carbon yield with different adsorbent/catalyst ratios.

DME yield calculations according to eq 35, which also include the blowdown contribution, result in a similar trend (Figure 6) to that reported in the literature by van Kampen et al.,^46^ with a maximum just above 70% at 8/1 w/w adsorbent/catalyst ratio. Notably, in the case of 2/1 w/w ratio, the lack of adsorbent results in a marked DME yield decrease due to the negative thermodynamic impact of the higher water load on DME production (as also shown in Figure 3). The yield at 16/1 w/w ratio, on the contrary, is slightly lower than the maximum one because of kinetic effects associated with the thermal behavior described in the following.

**Figure 7:**
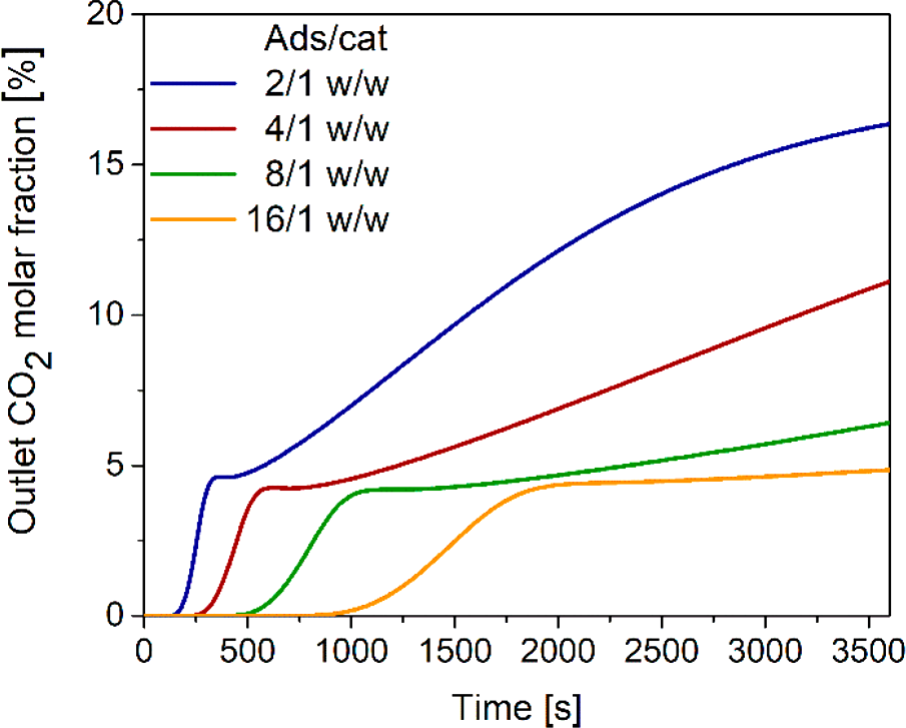
Time evolution of outlet CO_2_ molar fraction with different adsorbent/catalyst weight ratios.

**Figure 8:**
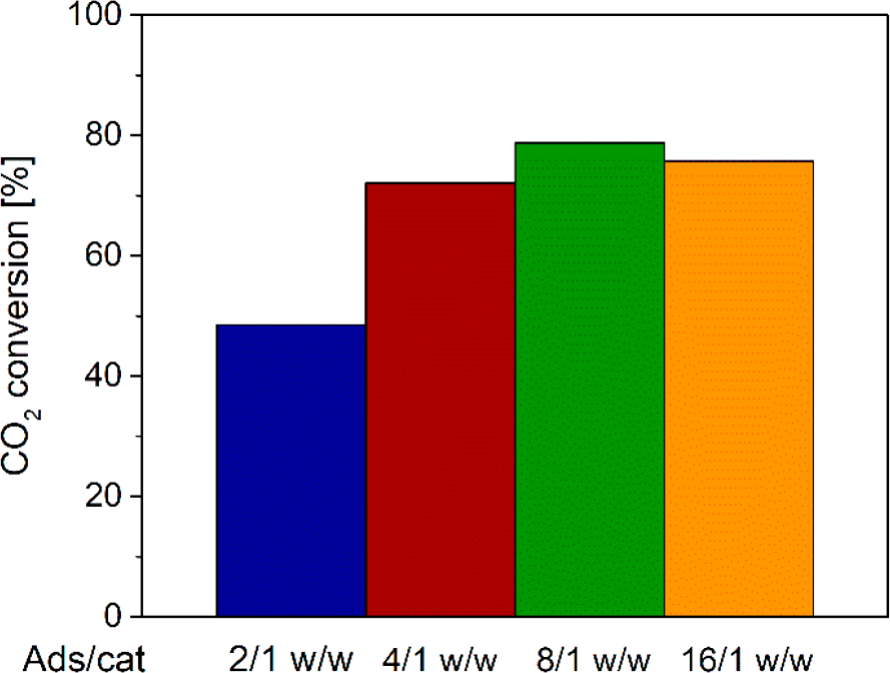
CO_2_ conversion with different adsorbent/catalyst weight ratios.

Another important performance parameter to be considered is the outlet molar fraction of CO_2_. The SEDMES is indeed a process that might be adopted for carbon dioxide valorization:^26^ the conversion of CO_2_ is therefore one of the key targets. Figure 7 shows that, after the breakthrough, the outlet CO_2_ molar fraction increases faster when the adsorbent to catalyst weight ratio is smaller. This is due to the negative thermodynamic effect of the increasing water hold up on the adsorbent. As a result, as shown in Figure 8, the overall CO_2_ conversion calculated according to eq 37 increases with the adsorbent to catalyst ratio up to 8/1 w/w and slightly decreases at 16/1 w/w mirroring the trend calculated for the DME yield.

**Figure 9:**
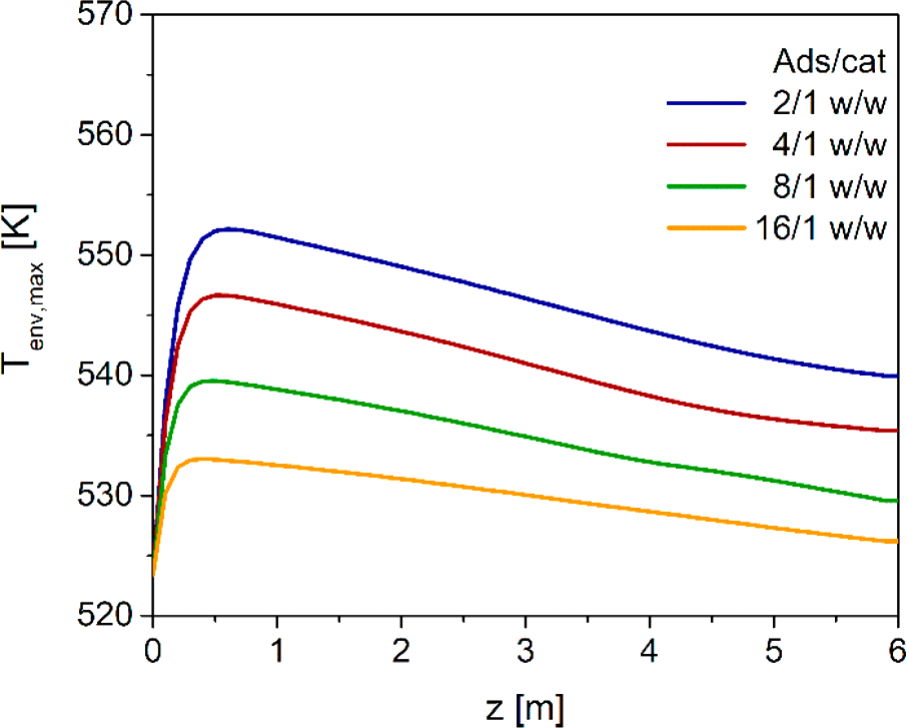
Axial envelope of maximum local temperatures with different adsorbent/catalyst weight ratios.

Finally, thermal aspects can be analyzed by looking at the envelopes of maximum temperatures calculated for the different adsorbent/catalyst ratio (Figure 9). Results obtained in a previous paper by the authors^50^ evidenced that the higher exothermicity of SEDMES process compared with the conventional DME direct synthesis, caused by the additional heat released by H_2_O adsorption (see eqs 5 and 6), is compensated by the catalyst dilution with the adsorbent. This allows to use tube diameters up to 38 mm with a 4/1 w/w adsorbent to catalyst ratio.^50^Figure 9 shows that the dilution effect increases with the adsorbent/catalyst ratio, so that the thermal stress of the catalyst is higher for less diluted beds. However, even with the lowest adsorbent to catalyst ratio herein considered (2/1 w/w), the temperature level is not that critical, considering that the maximum temperature is at 552 K, i.e., still below both the threshold limits of 553 and 573 K reported in the literature^23,74^ to prevent the deactivation of CZA catalyst in methanol synthesis conditions. On the other hand, a very mild profile of the envelope of maximum temperatures is obtained at the highest adsorbent/catalyst ratios, which limits the reaction kinetics and is responsible for the decrease in both the DME yield and the CO_2_ conversion observed in Figures 6 and 8, respectively.

As reported in van Kampen et al.,^46^ for an optimal design of the SEDMES process, the productivity vs yield/conversion trade off evidenced by comparing Figures 4 and 6 needs to be adjusted by properly setting the cycle regeneration and adsorption/reaction steps.^39,46^

### 3.2 Effect of Catalyst Pellet Diameter

The results reported in the previous section show that the adsorbent/catalyst ratio controls both the capacity of the system to remove water and the maximum temperature profile. The other main factor influencing the SEDMES process is the kinetic of DME production, which is potentially affected by the diffusion limitation inside the catalyst pellets. As stated in the introduction, the diffusion limitations occurring when using the mechanical mixture of different pellets of MeOH and DME catalysts in the conventional direct DME synthesis significantly hinder the apparent reaction rates.^51,52,55^

Notoriously, the simplest way to minimize the internal diffusion limitations consists in reducing the catalyst particle diameter.^55^ In order to check the influence of diffusion effects on the SEDMES process, the pellet diameter of both MeOH and DME catalysts is reduced in the following from 3 mm to 1.5 and 1 mm. Simulations are performed with a 4/1 w/w adsorbent/catalyst ratio.

**Figure 10:**
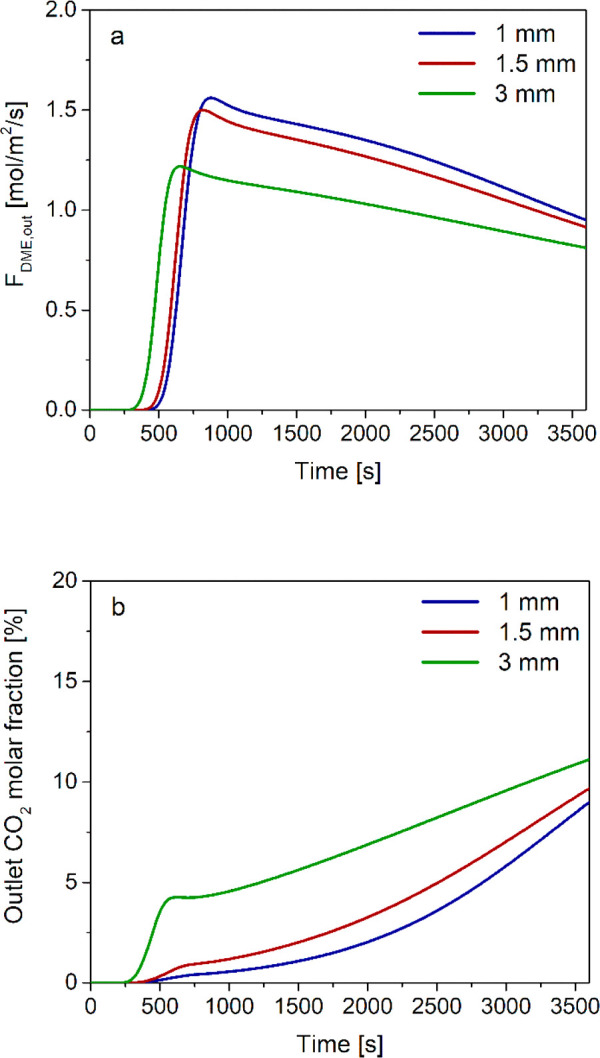
Time evolution of (a) outlet DME specific flow rate and (b) outlet CO_2_ molar fraction with different catalyst pellet diameters and adsorbent/catalyst = 4/1 w/w.

The time evolution of outlet DME specific flow rate for different catalyst pellet diameters is reported in Figure 10a. The production of DME significantly increases on reducing the pellet diameter, evidencing that intraparticle diffusion limitations have a significant impact on reactor performances also in SEDMES. The delayed breakthrough occurring on decreasing the diameter is a consequence of the volumetric flow rate reduction caused by the gas phase molar contraction associated with the higher production of DME according to stoichiometries 5 and 6. Upon the maximum, the outlet flow rate decreases with a slightly steeper slope on decreasing the pellet diameters, which is due to higher adsorbent hold-up.

The gain in terms of overall DME carbon yield is significant (Table 8): the calculated yield grows from 66.7% with a diameter of 3 mm to 79.7% with a pellet diameter of 1 mm. Note that at fixed adsorbent/catalyst ratio and constant GHSV, the DME productivity is strictly proportional to the yield. The improved effective reaction rates also increase CO_*x*_ and CO_2_ conversions. Specifically, the carbon dioxide breakthrough is delayed and far less sharp than in the standard case on decreasing the pellet size (Figure 10b). The overall CO_2_ conversion increases from 72.1% at *d*_p_ = 3 mm to 85.3% at *d*_p_ = 1 mm (Table 8).

**Table 8:** DME Carbon Yield, CO_2_ and CO_*x*_ Conversion and DME productivity with Different Catalyst Pellet Diameters and Different Catalyst Pellet Configuration (Mechanical Mixture, Hybrid, MeOH@DME and DME@MeOH Core@Shell)

		mechanical mixture			hybrid	MeOH@DME		DME@MeOH
ads/cat	kg/kg	1/1	1/1	1/1	1/1	1/1	2/1	1/1
*d*_p_	mm	1	1.5	3	3	3	3	3
*Y*_DME_	%	79.7	76.8	66.6	76.4	70.8	75.2	75.3
Conv_CO_2__	%	85.3	82.5	72.1	80.0	71.2	77.4	81.8
Conv_COx_	%	81.4	78.6	68.1	78.4	72.6	76.8	77.5
Prod_DME_	kg/(h m^3^)	30.6	29.5	25.6	29.4	27.2	28.9	28.9

**Figure 11:**
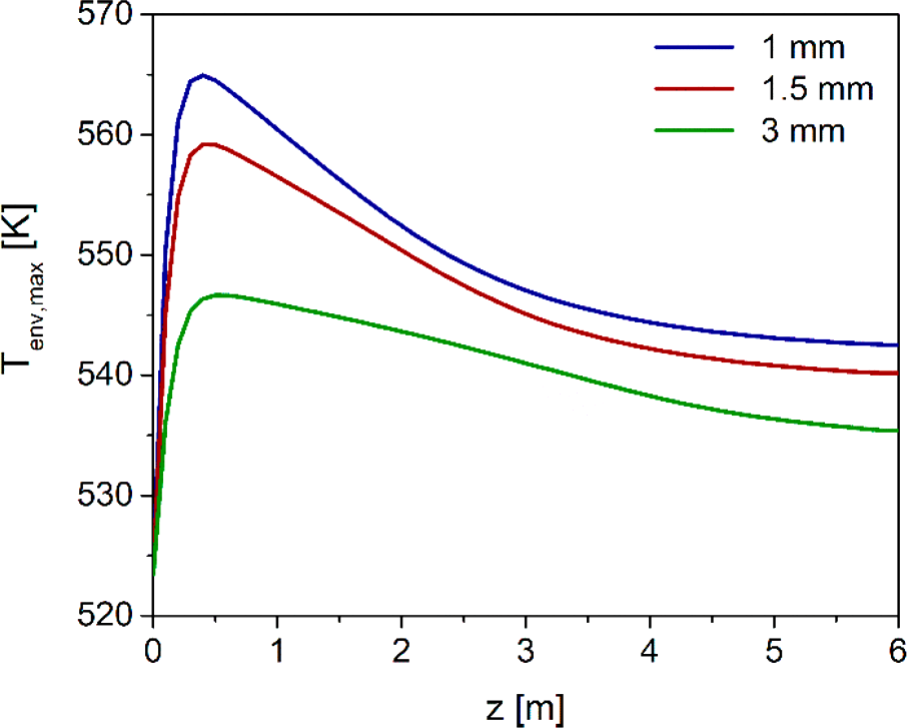
Axial envelope of maximum local temperatures with different catalyst pellet diameters.

On the other hand, the faster DME production with small diameter pellets results in an increase in the temperature level along the axial coordinate (Figure 11). The temperature hot spot in first part of the adsorbent/catalyst bed becomes significantly more pronounced, coherently with the increment in reactants conversion. The temperatures reached with *d*_p_ ≥ 1.5 mm exceed the lowest safety threshold of 553 K, which might cause catalyst deactivation problems.

These results highlight that is possible to improve the DME yield by reducing the catalyst pellet diameter. However, for industrial scale reactors, this may lead to an unacceptable increase of the pressure drops, and pellets of at least few millimeters of diameter must be adopted.^17,51,67^

It may be argued that the typical values of specific flow rates used in the adsorption/reaction step of SEDMES process are smaller by a factor of 5–10 with respect to those used in conventional dimethyl ether or methanol synthesis:^17,67^ this would make the pressure drops in the adsorption/reaction step limited even when using 1 mm catalyst pellets. However, a larger specific flow rate is preferable in the regeneration steps: incrementing the purge flow rate results indeed in a more efficient adsorbent regeneration.^46^ This constraint still makes adoption of small particle diameter a critical issue. Accordingly, in the next section, the catalyst coupling in hybrid or core@shell pellets is considered as an alternative to particle size reduction for minimizing the impact of intraparticle diffusion limitations without affecting the pressure drops.

### 3.3 Effect of Active Phase Distribution

In the hybrid pellets, the two catalysts are intimately coupled (see Figure 1), allowing for reducing the diffusion lengths without changing the catalyst particle diameter.^51^

**Figure 12:**
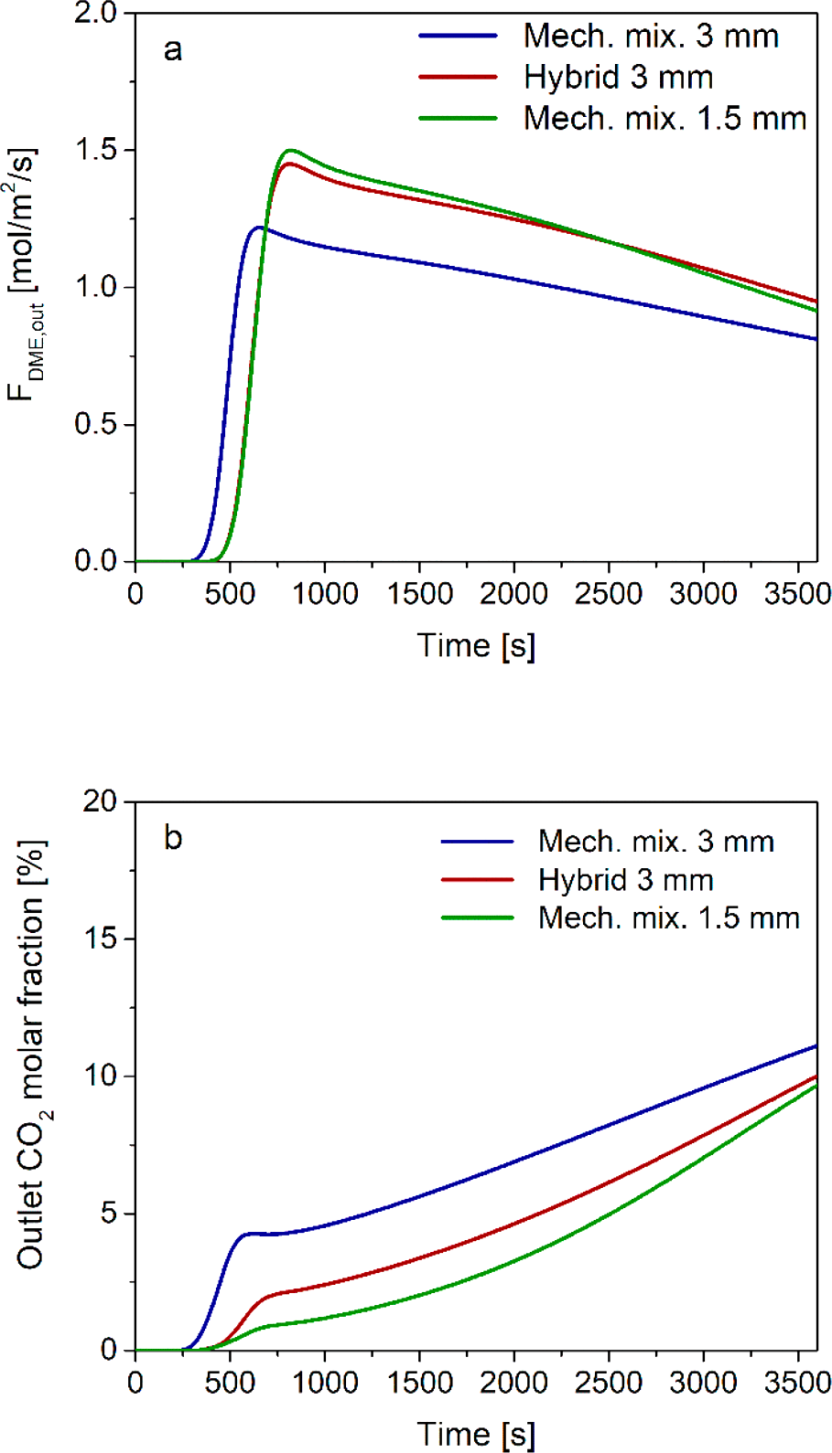
Time evolution of the (a) outlet DME specific flow rate and (b) outlet CO_2_ molar fraction with mechanical mixture of different catalyst pellets (MeOH and DME) with particle diameters of 1.5 and 3 mm, and hybrid pellets with particle diameter 3 mm.

In Figure 12a, the mechanical mixture and hybrid pellet configurations are compared in terms of specific outlet DME flow rate. Simulation results show that the hybrid pellets with 3 mm diameters produce higher amounts of DME than a mechanical mixture of pellets with the same diameter, with the outlet DME flow rate being similar to the one obtained with 1.5 mm pellets. This is confirmed by the overall DME yield reported in Table 8: the hybrid configuration significantly outperforms the mechanical mixture with the same pellet size (76.4% vs 66.6%), closely approaching the mechanical mixture with 1.5 mm particles.

By adopting the hybrid catalyst layout, it is therefore possible to achieve yield performances comparable to those obtained by using small diameter pellets to reduce diffusional limitations in the pores.^55^ This does not mean that the catalytic pellet behavior in the two cases is exactly the same: the improvement in DME yield is given by the reduction of the diffusion lengths in the case of smaller particle diameter, whereas the advantage of hybrid configuration is that the active phase segregation is eliminated, synergistically coupling the methanol synthesis and dehydration from both a kinetic and a thermodynamic point of view.^53^ Indeed, methanol is produced in the same place where it is dehydrated to DME, which thermodynamically promotes methanol synthesis by action of mass and kinetically enhances the dehydration reaction by reducing the intraparticle diffusion resistances.^51^

The different behavior of hybrid pellets with larger diameters and a mechanical mixture of small (1.5 mm) pellets is more evident when focusing on the outlet CO_2_ concentration trajectories reported in Figure 12b. While producing almost the same amount of DME, the outlet CO_2_ molar fraction grows more rapidly when using hybrid pellet instead of the mechanical mixture with 1.5 mm particles, resulting in a lower value of CO_2_ overall conversion (Table 8). In fact, in hybrid pellets, water is locally produced by both methanol synthesis (from CO_2_) and dehydration catalysts, thus shifting the thermodynamic equilibrium of the WGS/r-WGS and resulting in a low net rate of CO_2_ consumption. On the contrary, in the mechanical mixture, methanol synthesis and methanol dehydration occur on different pellets, thus resulting in a lower H_2_O concentration in the pores of the methanol catalyst, which is responsible for WGS/r-WGS equilibration.

**Figure 13:**
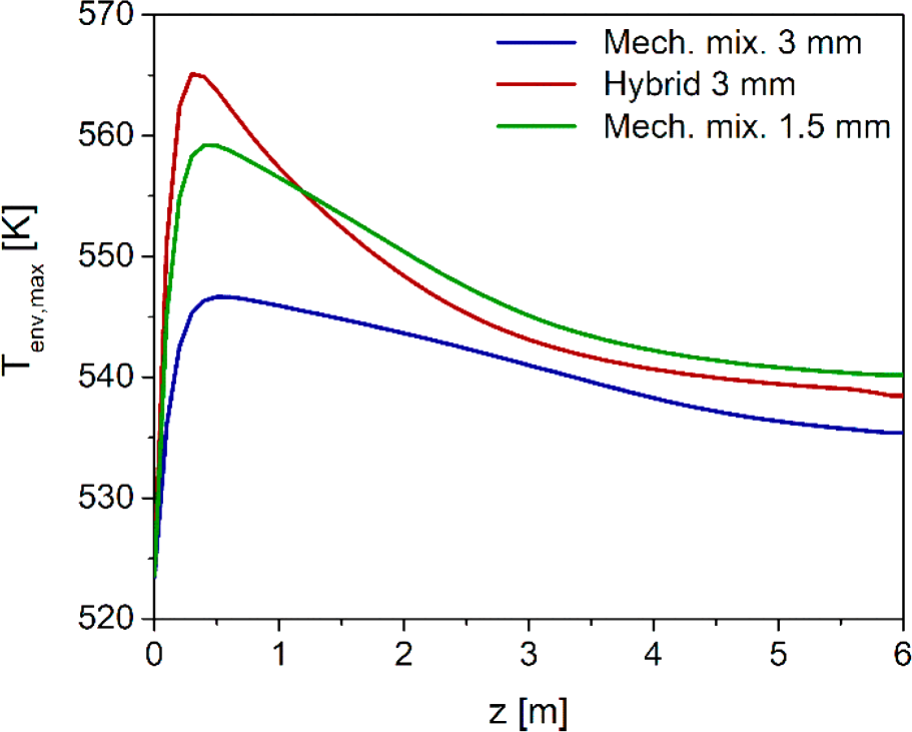
Axial envelope of maximum local temperatures with mechanical mixtures of different catalyst pellets (MeOH and DME) with particle diameters of 1.5 and 3 mm, and hybrid pellets with particle diameter 3 mm.

**Figure 14:**
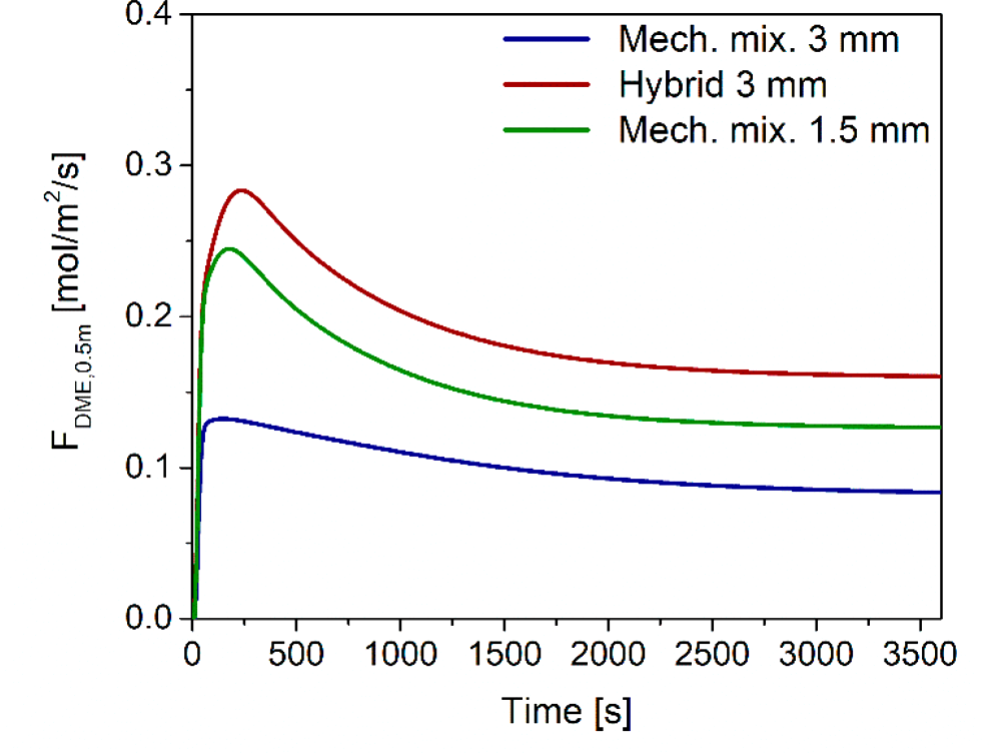
Time evolution of the DME specific flow rate at a 0.5 m axial position with mechanical mixtures of different catalyst pellets (MeOH and DME) with particle diameters of 1.5 and 3 mm, and hybrid pellets with particle diameter 3 mm.

The different behavior of the hybrid pellet and mechanical mixture of small diameter pellets is also evident from the inspection of the envelopes of maximum temperature plotted in Figure 13. With the hybrid pellets, the maximum temperature shows a pronounced peak (565 K) in the first part of the fixed bed and then decreases sharply, crossing at *z* ≈ 1 m the envelope of maxima of the mechanical mixture with *d*_p_ = 1.5 mm, which exhibits a milder peak (559 K). This occurs because the spatial distribution of reactant conversion to DME is not the same. Time evolution of the specific DME flow rate at 0.5 m, which is just after the maximum temperature hot spot, shows that the hybrid pellet configuration promotes a faster DME production in the first part of the reactor (Figure 14). On the other hand, in the case of the mechanical mixture with *d*_p_ = 1.5 mm, the reactions take place more evenly along all the axial coordinate, resulting in DME flow rate trajectories that are almost overlapped at the reactor outlet (Figure 12a).

Notably, the maximum experienced temperature for the hybrid pellet configuration significantly exceeds the lowest safety threshold temperature of 553 K reported in the literature.^23^ Although the hottest temperatures are experienced in only a fraction of the reaction/adsorption phase, this may expose the methanol synthesis catalyst to the risk of long-term deactivation due to Cu sintering.

Moreover, it is reported that in hybrid pellets, deactivation may also occur because of the close interaction between the two active phases, possibly leading to detrimental cross migration of elements at the boundaries.^75^ The core@shell catalysts are proposed as alternative to the mechanical mixture and the hybrid configurations, as the diffusion lengths are significantly reduced^51,54,55^ while allowing for a better temperature control.^51^ Besides, the core@shell design significantly reduces the contact area between the active phases, partially preventing the aforementioned deactivation.^59^ In addition, an intermediate protective layer can be added between the core and the shell, further reducing the detrimental interactions.^65^

In view of this, both the core@shell configurations sketched in Figure 1, with methanol synthesis (MeOH@DME) or methanol dehydration catalyst (DME@MeOH) in the pellet core, are simulated. It is important to remark that, due to the different density of CZA and γ-Al_2_O_3_ (Table 4), the internal interface radius *r*_int_ (calculated as reported in eqs 26b and 26c) is different in the two core@shell configurations even if the same MeOH/DME ratio is considered. Assuming pellet diameter of 3 mm with a MeOH/DME ratio 1/1 w/w, *r*_int_ = 1.12 mm for MeOH@DME and *r*_int_ = 1.23 mm for DME@MeOH are obtained.

**Figure 15:**
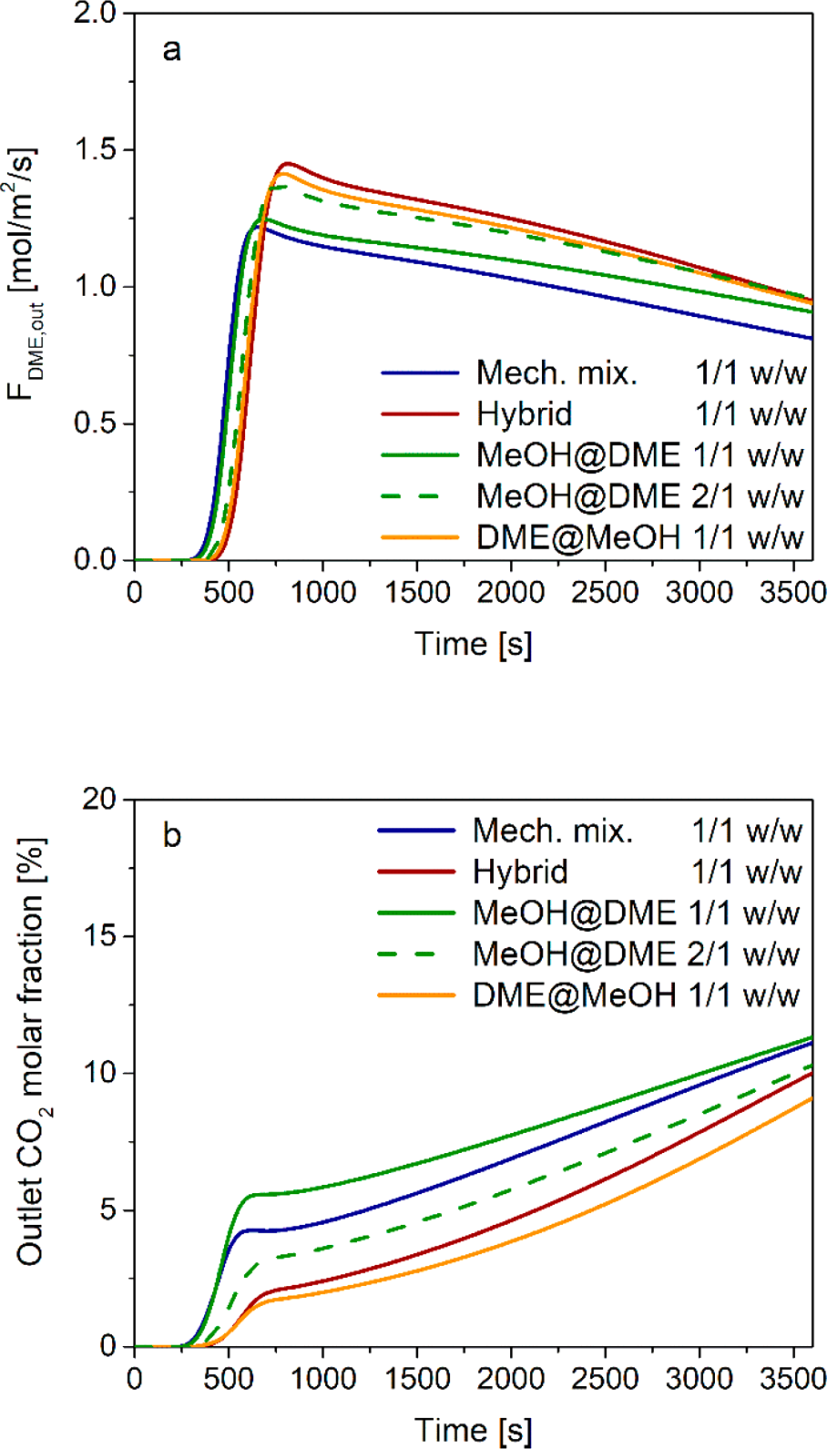
Time evolution of the (a) outlet DME specific flow rate and (b) outlet CO_2_ molar fraction with different catalyst pellet configurations (mechanical mixture, hybrid, MeOH@DME, DME@MeOH).

The results of core@shell configurations are compared with those of the mechanical mixture and the hybrid pellet taken as benchmark. The calculated DME outlet specific flow rates are reported in Figure 15a. The hybrid configuration maximizes the production of DME, closely followed by the DME@MeOH (showing a similar profile), whereas the MeOH@DME initially behaves similarly to the mechanical mixture but then decreases more gradually. Thus, the ranking of the DME carbon yield and CO_*x*_ conversion is hybrid > DME@MeOH > MeOH@DME > mechanical mixture (Table 8).

Focusing on CO_2_ conversion, the analysis of outlet concentration trajectories in Figure 15b and conversion data in Table 8 provide a slightly different ranking: DME@MeOH > hybrid > MeOH@DME > mechanical mixture.

These results resemble those obtained in the conventional direct DME synthesis for the 1/1 w/w MeOH/DME catalyst ratio^51^ and can be explained on the basis of the specific characteristics of the active phase distributions in the different pellet configurations. In fact, DME@MeOH pellets minimize the diffusion length in methanol synthesis catalyst, promoting the production of methanol at the expense of the selectivity to DME, and better performing with low MeOH/DME catalyst ratios. This configuration is even more advantageous in SEDMES conditions, where the DME selectivity is less critical^26^ thanks to the positive effect of in situ H_2_O removal on the dehydration kinetics, and the process is mainly limited by the methanol production.

On the other hand, the MeOH@DME configuration strongly reduces the diffusion length of the DME catalyst, thus maximizing the selectivity to DME. Accordingly, the production of methanol becomes the limiting factor of this configuration, which performs better with MeOH/DME catalyst ratios larger than 1/1 w/w.^51^

To further assess this aspect, we performed simulations considering MeOH@DME pellets with a catalyst ratio MeOH/DME = 2/1 w/w (MeOH core radius = 1.26 mm). Results in Figure 15 and Table 8 confirm that a significant performance increase is obtained, closely approaching the DME yield and CO_*x*_ and CO_2_ conversion obtained with hybrid pellets.

**Figure 16:**
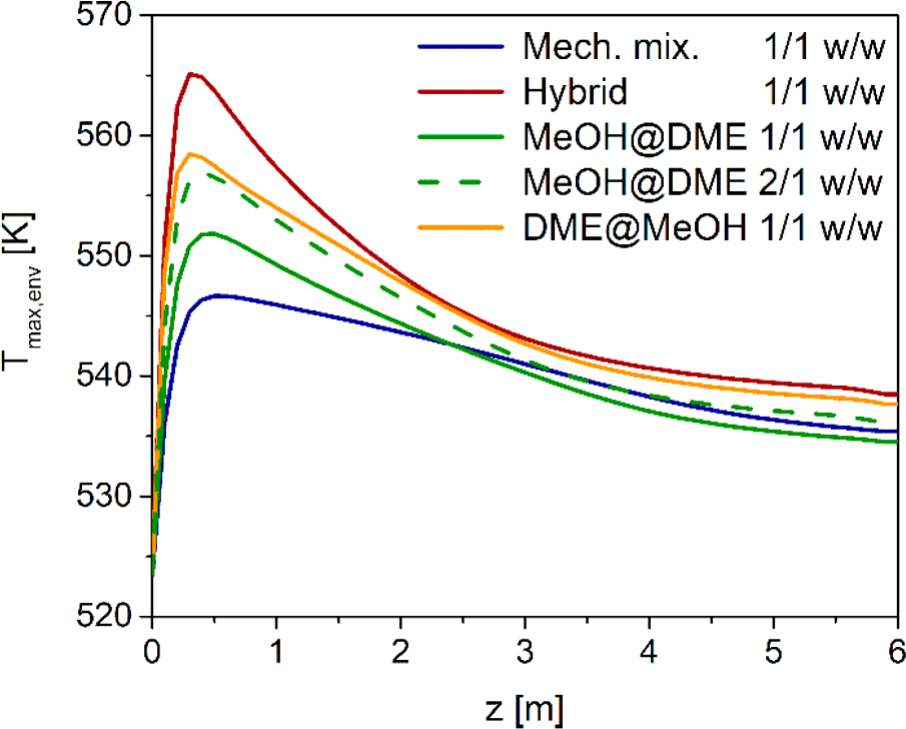
Axial envelope of maximum local temperatures with different catalyst pellet configurations (mechanical mixture, hybrid, MeOH@DME, DME@MeOH).

Finally, the axial envelopes of local maximum temperature obtained with different MeOH/DME active phase distributions are shown in Figure 16. The profiles are consistent with the DME flow rates in Figure 15a. The highest hot spot temperatures are obtained with the hybrid pellet, whereas the mechanical mixture is the least thermally stressed configuration. The core@shell pellets have intermediate maximum temperatures: the DME@MeOH has the maximum hot spot of 558 K, just below the hybrid configuration (565 K), whereas with the MeOH@DME, a maximum temperature of 551 K, moderately higher than that of the mechanical mixture (546 K) is reached, which increases to 557 K when MeOH/DME = 2 w/w is used.

This trend differs from that obtained for the conventional DME synthesis,^51^ in which the MeOH@DME secures a good thermal control while having relatively high DME yields (comparable to the hybrid). Indeed, the exothermicity of the direct DME synthesis is mostly related to the methanol synthesis reaction and the MeOH@DME catalyst, despite a methanol production lower than the DME@MeOH and hybrid, has the highest selectivity to DME, resulting in milder hot spots. In the present case, however, the exothermicity of the process is merely connected to the conversion of reactants to DME. The advantage of MeOH@DME is indeed lost in the SEDMES process, which shows extremely high selectivity to DME independently of the catalyst configuration considered.

In conclusion, the DME@MeOH core@shell catalyst configuration seems the most promising for SEDMES, as a DME yield comparable to the hybrid (the maximum one) is obtained simultaneously with an improved CO_2_ consumption and a better thermal control.

### 3.4 Effect of Operating Variables

A techno-economic analysis recently reported in the literature^41^ shows that installation costs of the SEDMES unit play a significant role in DME production costs. Results in the previous section point out that catalyst distribution at the pellet scale may significantly improve the DME yield/productivity performances of the reactor. To better assess the potential of hybrid and core@shell pellets for process intensification, in this section, we investigate the effect of space velocity and pressure on reactor performances.

Simulations are first performed by doubling the GHSV_cat_ from 805 to 1610 h^–1^ considering the mechanical mixture configuration with a 4/1 w/w adsorbent/catalyst ratio and the standard 1/1 w/w MeOH/DME catalyst weight ratio. Referring to the whole bed volume, these GHSVs correspond to 140 and 280 h^–1^, which are values within the range reported in the literature SEDMES techno-economic analysis.^41^ Results reported in Tables 8 and 9 show that by doubling the GHSV, the productivity grows from 25.6 to 32.5 kg_DME_/(h m^3^). However, the DME yield, as well as CO_2_ and CO_*x*_ conversions, drops by more than 20%.

**Table 9:** DME Carbon Yield, CO_2_ and CO_*x*_ Conversion and DME Productivity (GHSV_cat_ = 1610 h^–1^) with Different Catalyst Pellet Configuration (Mechanical Mixture, Hybrid, DME@MeOH), Pressure, and Tube Diameter

		mechanical mixture		hybrid pellet			DME@MeOH	
*P*^0^	bar	25	50	25	50	50	50	50
GHSV_cat_	h^–1^	1610	1610	1610	1610	1610	1610	1610
*d*_t_	mm	38	38	38	38	25.6	38	25.6
*Y*_DME_	%	42.3	66.7	52.9	72.2	72.3	74.8	75.7
Conv_CO2_	%	40.9	64.8	45.2	69.7	70.1	74.2	75.6
Conv_COx_	%	45.5	71.6	56.1	76.5	77.2	78.5	80.1
Prod_DME_	kg/(h m^3^)	32.5	51.3	40.7	55.5	55.6	57.5	58.2

When considering the hybrid pellet configuration operated at 1610 h^–1^, despite the better productivity (Table 9), DME yield is still too low (52.9%) for economical sustainability.

The issue of low DME yield can be overcome by increasing the pressure from 25 to 50 bar, which kinetically and thermodynamically favors the DME direct synthesis process and enhances the water adsorption capacity.^45^ Simulations results reported in Table 9 show that DME yields of 66.7 and 72.2% are obtained at 50 bar with the mechanical mixture and the hybrid pellet configuration, respectively, with corresponding productivities of 51.3 and 55.5 kg/(h m^3^), more than two times higher than in the base case (mechanical mixture, GHSV_cat_ = 805 h^–1^, *P* = 25 bar).

The DME@MeOH catalyst configuration, which in the previous section was identified as the best core@shell configuration, is also simulated, showing performances slightly better than the hybrid pellet at high pressure and high GHSV (Table 9). The reason of this behavior can be explained considering that the high partial pressure of water detrimentally affects the methanol synthesis kinetics and thermodynamics. In the shell of DME@MeOH, where methanol synthesis occurs, the water partial pressure is lower than that inside the hybrid pellet, where water is produced both by methanol synthesis and dehydration.

**Figure 17:**
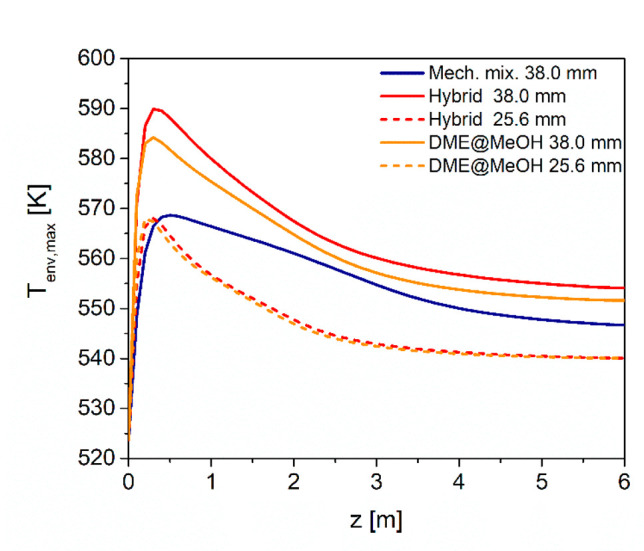
Axial envelope of maximum local temperatures at 50 bar with different catalyst pellet configurations (mechanical mixture, hybrid, DME@MeOH) and different tube diameters.

Consistently with the productivity enhancement, the temperature envelope grows drastically with the pressure (Figure 17), keeping below the highest literature threshold^74^ only with the mechanical mixture (maximum at 569 K). In fact, for the hybrid pellet and DME@MeOH configurations, maxima at 590 and 584 K are obtained, respectively, which significantly exceed the highest safety limit of 573 K.

A way to achieve a better temperature control consists in reducing the tube diameter. For this analysis, the tube diameter is reduced from 38.0 mm to 25.6 mm, still assuming high space velocity and pressure (1610 h^–1^, 50 bar) and considering both the hybrid and the DME@MeOH configurations. Figure 17 shows that the thermal stresses are effectively moderated by the reducing tube diameter to 25.6 mm, reaching a maximum temperature of 568 K for both the hybrid and the DME@MeOH catalysts.

Noteworthy, the tube diameter has a minor effect on all the performance indicators (Table 9): the DME yield, as well as the CO_2_ and CO_*x*_ conversions, increases less than 1% by decreasing from 38 to 25.6 mm tube diameter, independently from the catalyst configuration. This confirms that the DME@MeOH core@shell configuration is particularly interesting, as it provides yield performances comparable to or even better than hybrid pellets.

The results reported above on the effect of both the pellet configuration and the operating window of a SEDMES reactor, could provide a basis for the refinement of the techno-economic analysis of the DME production process,^41^ which is needed to fully assess the potential of the process intensification options herein investigated.

## 4 Conclusions

The conversion/yield/productivity performances and the thermal behavior of a full scale, externally cooled, fixed-bed, multitubular SEDMES reactor are assessed in this work by means of a 2D+1D model of a single catalytic tube, which simulates the adsorption/reaction step of a PSA cycle. The effects of adsorbent/catalyst ratio, catalyst particle diameter, active phase distribution (mechanical mixture, hybrid, and core@shell pellets), and operating variables (GHSV and pressure) are addressed.

Although DME productivity grows on decreasing the adsorbent/catalyst ratio, higher adsorbent/catalyst ratios increase the water adsorption capacity of the system, controlling the water breakthrough and improving both the CO_2_ conversion and the DME yield because of thermodynamic reasons. Besides, the adsorbent acts as a thermal diluent, thus allowing for a more effective temperature control of the reactor on increasing the adsorbent/catalyst ratio. A trade-off is needed, which requires the optimization of the full reaction adsorption/regeneration cycle in the framework of a techno-economic analysis.

Simulation results also show that, as for the conventional DME direct synthesis, the apparent reaction rates are hindered by catalyst intraparticle diffusion limitations. Accordingly, CO_2_ conversion, as well as DME yield and productivity, can be improved by reducing the catalyst pellet size, which, however, may result in unacceptable pressure drop. Besides, it should be considered that the maximum temperature reached in the reactor with smaller catalyst particles increases as a consequence of the larger heat released by reaction/adsorption.

The coupling of the methanol synthesis and dehydration catalysts in a single hybrid pellet is a possible solution to pore diffusion limitations, without the need to reduce the particle diameter. Our results show that DME yield and productivity similar to those achieved with the mechanical mixture of 1.5 mm pellets are obtained with 3 mm hybrid pellets. Only the CO_2_ conversion is slightly lower, because of the increased water concentration inside the catalyst pellets. Nevertheless, the adoption of hybrid pellets has been reported to cause deactivation problems because of the interaction between MeOH and DME active phases.

The core@shell catalysts (MeOH@DME and DME@MeOH) are therefore proposed as trade-off to reduce the intraparticle diffusion lengths while allowing a lower contact surface between the different active phases. At SEDMES conditions, the DME@MeOH configuration is particularly promising, granting DME yield and CO_2_ conversion comparable to those obtained with hybrid pellets while moderating the maximum temperature in the reactor and increasing the catalyst stability.

Finally, our results indicate that the performance of the SEDMES process can be improved by increasing the space velocity (resulting in higher DME productivity) and the pressure (resulting in higher DME yield and productivity). This results, however, in a more difficult heat management in the reactor, which requires smaller tube diameters to avoid the catalyst temperature markedly exceeding the safety limits.

## Supporting Information Available

Transport correlations and physical properties; reaction kinetics constants; adsorption isotherm (PDF)

## Dedication

We dedicate this article to Enrico Tronconi in gratitude for his scientific mentorship on the occasion of his 65th birthday

## Notation

*a*_v_: Solid specific surface area per unit volume (m^2^/m^3^)
*b*: Adsorption equilibrium constant
*C*_p_: Gas mixture specific heat (J/(kg K))
C_i_: Molar concentration of species *i* (mol/m^3^)
*C*_tot_: Total molar concentration (mol/m^3^)
Conv_CO*x*_: CO_*x*_ conversion (%)
*d*_p_: Pellet diameter (m)
*d*_t_: Tube internal diameter (m)
*D*_ae,*i*_: Effective axial dispersion of species *i* (m^2^/s)
*D*_eff,*i*_: Effective diffusion coefficient of species *i* in solid (m^2^/s)
*D*_re,*i*_: Effective radial dispersion of species *i* (m^2^/s)
*D*_*ij*_: Binary diffusion coefficient of species *i* in species *j* (m^2^/s)
*D*_k,*i*_: Knudsen diffusion coefficient of species *i* (m^2^/s)
*D*_mix,*i*_: Molecular diffusion coefficient of species *i* (m^2^/s)
*f*_*i*_: Fugacity of species *i* (bar)
*F*_*i*_: Molar flow rate of species *i* per unit area (mol/m^2^/s)
*F*_tot_: Total molar flow rate per unit area (mol/(m^2^ s))
*h*_gs_: Gas–solid heat transfer coefficient (W/(m^2^ K))
*h*_w_: Wall heat transfer coefficient (W/(m^2^ K))
*h*_w,conv_: Wall convective heat transfer coefficient (W/(m^2^ K))
*k*_1_: Kinetic constant of CO hydrogenation to methanol (mol/(kg_cat_ s bar^3/2^))
*k*_2_: Kinetic constant of reverse water gas shift (mol/(kg_cat_ s bar))
*k*_3_: Kinetic constant of CO_2_ hydrogenation to methanol (mol/(kg_cat_ s bar^3/2^))
*k*_4_: Kinetic constant of methanol dehydration to dimethyl ether (mol/(kg_cat_s))
*k*_m,*i*_: Gas–solid mass transfer coefficient of species *i* (m/s)
K_CH_3_OH_: Adsorption constant of methanol on dehydration catalyst (m^3^/mol)
*K*_CO_: Adsorption constant of CO on methanol synthesis catalyst (bar^–1^)
*K*_CO_2__: Adsorption constant of CO_2_ on methanol synthesis catalyst (bar^–1^)
*K*_eq*,**j*_: Equilibrium constant of reaction *j*
*K*_H_2_O_: Adsorption constant of H_2_O on dehydration catalyst (m^3^/mol)
*K*_H_2_O/H_2__: Adsorption group of H_2_O/H_2_ on methanol synthesis catalyst (bar^–1/2^)
*K*_LDF_: Linear driving force coefficient (s^–1^)
*L*_t_: Tube length (m)
MW_*i*_: Molar weight of species *i* (kg/mol)
*N*_C_: Number of components
*N*_cat_: Number of catalyst phases
*N*_R_: Number of reactions
*Nu*: Nusselt number
*P*: Pressure (Pa)
*Pr*: Prandtl number
Prod_DME_: DME productivity (kg_DME_/(h m^3^))
*q*: Adsorbent water load (mol/kg)
*q*_s_: Saturated adsorption capacity (mol/kg)
*q*_sat_: Adsorbent saturation water load (mol/kg)
*r*: Reactor radial coordinate (m)
*r*_p_: Pellet radius (m)
*r*_pore_: Pore radius (m)
*R*: Gas universal constant (J/(mol K))
*R*_*j*_: Rate of reaction *j* (mol/(kg s))
*Re*: Reynolds number
*S*_p_: Geometric pellet surface area (m^2^)
*Sc*: Schmidt number
*Sh*: Sherwood number
*t*: Time (s)
*T*: Temperature (K)
*T*_cool_: Coolant temperature (K)
*v*_gas_: Gas velocity (m/s)
*V*: Volume (m^3^)
*x*: Pellet radial coordinate (m)
*y*_*i*_: Molar fraction of species *i*
*Y*_DME_: Dimethyl ether carbon yield
*z*: Reactor axial coordinate (m)
**Greek letters**
Δ*H*_ads_: Heat of H_2_O adsorption (J/mol)
Δ*H*_r,*j*_: Heat of reaction *j* (J/mol)
ε_b_: Bed void fraction
ε_p_: Particle porosity
η_j_: Catalyst effectiveness factor of reaction *j*
λ_ax_: Effective axial thermal conductivity (W/(m K))
λ_rad_: Effective radial thermal conductivity (W/(m K))
λ_gas_: Gas mixture thermal conductivity (W/(m K))
λ_s_: Solid thermal conductivity (W/(m K))
ν_ij_: Stoichiometric coefficient of species *i* in reaction *j*
ξ_ads_: Volumetric fraction of adsorbent (*V*_ads_/*V*_s_)
ξ_cat_: Volumetric fraction of catalyst (*V*_cat_/*V*_s_)
ρ: Density (kg/m^3^)
τ: Tortuosity
υ_i_: Diffusional volume of species *i* (cm^3^/mol)
ψ_cat_*k*__: Volumetric fraction of *k*-catalyst phase (*V*_cat_*k*__/*V*_cat_)
**Superscripts and subscripts**
0: Reactor inlet condition
ads: Adsorbent phase
ae: Effective axial
av: Average
ax: Axial
b: Bed
cat: Catalyst phase
cat_*k*_: *k*-catalyst phase (*k* = MeOH, DME)
cool: Coolant
eff: Effective
end: End of reaction/adsorption step
gas: Gas phase
*i*: *i*-species (*i* = CO, CO_2_, H_2_, H_2_O, MeOH, DME, N_2_)
int: Core@shell pellet interface
*j*: *j*-reaction
p: Internal pellet
pa: Spherical particle with equal surface area
pe: Equivalent spherical particle
pv: Spherical particle with equal volume
rad: Radial
re: Effective radial
s: Solid phase
surf: Catalyst pellet surface
t: Tube
tot: Total
w: Tube wall
