# Supplementary material for: Model Analysis of the Role of Kinetics, Adsorption Capacity, and Heat and Mass Transfer Effects in Sorption Enhanced Dimethyl Ether Synthesis
Source: Ind Eng Chem Res. 2021 Mar 23;60(18):6767–83. doi: 10.1021/acs.iecr.1c00521 (PMC8154431; doi:10.1021/acs.iecr.1c00521)
Supplement: Supplementary file 1 — ie1c00521_si_001.pdf [file ie1c00521_si_001.pdf]

Supporting information

Model analysis of the role of kinetics, adsorption capacity, heat and mass transfer effects in sorption enhanced dimethyl ether synthesis

Simone Guffanti[a], Carlo Giorgio Visconti[a], Gianpiero Groppi[a]*

[a]Laboratory of Catalysis and Catalytic Processes, Dipartimento di Energia, Politecnico di Milano, via La Masa 34, 20156 Milano, Italy

*to whom correspondence should be addressed:

gianpiero.groppi@polimi.it

S1. Transport correlations and physical properties

S1.1 Mass and heat gas-solid transport correlations

The mass and heat transport coefficients are calculated as in equations (S1) and (S2), taken from literature reference[1].

$$k_{m,i} = \frac{D_{mix,i} Sh_i}{d_{pe}}$$

$$Sh_i = 1.26 Re_{d_{pe}} Sc_i^{1/3} \left(\left(\frac{1-(1-\varepsilon_b)^{5/3}}{2-3(1-\varepsilon_b)^{1/3}+3(1-\varepsilon_b)^{5/3}-2(1-\varepsilon_b)^2} \right)^{-0.5} Re_{d_{pe}} \right)^{-2/3} \quad \text{(S1)}$$

$$h_{gs} = \frac{\lambda_{gas} Nu}{d_{pe}}$$

$$Nu = 1.26 Re_{d_{pe}} Pr^{1/3} \left(\left(\frac{1-(1-\varepsilon_b)^{5/3}}{2-3(1-\varepsilon_b)^{1/3}+3(1-\varepsilon_b)^{5/3}-2(1-\varepsilon_b)^2} \right)^{-0.5} Re_{d_{pe}} \right)^{-2/3} \quad \text{(S2)}$$

S1.2 Mass and thermal dispersion correlations

The effective radial diffusivity is calculated as reported in equation (S3) with the correlation taken from[2]. Two different correlations, one for the catalyst beds and one for the adsorbent bed, are adopted for estimating the effective axial diffusivity. The axial effective diffusivity for adsorbent beds is used only for water, while the correlation for catalyst beds is used for all the other components. The two correlation are reported in equation (S4).

$$D_{er,i} = \varepsilon_b \left(D_{mix,i} \sqrt{\varepsilon_b} + 0.1 d_{pe}\, v_{gas} \right) \quad \text{(S3)}$$

$$D_{ae,ads,H_2O} = \frac{20}{\varepsilon_b} D_{mix,H_2O} + 0.5 v_{gas} d_{pe}$$

$$D_{ae,cat,i} = \frac{D_{mix,i}}{\tau} + 0.5 v_{gas} d_{pe} \quad \text{(S4)}$$

The gas mixture diffusivities (eq. S5) are calculated as in the reference[3].

$$D_{mix,i} = \left(\sum_{j=1, j\neq i}^{NC} \frac{y_j}{D_{ij}} \right)^{-1} \quad \text{(S5)}$$

The binary molecular diffusivities for each species (eq. S6) are averaged using the Fuller equation[4].

$$D_{ij} = \frac{0.0143\, T^{1.75}}{P \sqrt{\frac{2000}{\frac{1}{MW_i} + \frac{1}{MW_j}}} \left[(\sum v_i)^{1/3} + \left(\sum v_j\right)^{1/3} \right]^2} \tag{S6}$$

The effective radial conductivity is calculated with the correlation taken from[5] (eq. S7).

$$\frac{\lambda_{rad}}{\lambda_{gas}} = \varepsilon_b + \frac{1 - \varepsilon_b}{0.22\ \varepsilon_b{}^2 + \frac{2}{3}\left(\frac{\lambda_{gas}}{\lambda_s}\right)} + \frac{Re_{d_{pa}} Pr}{8.65 \left[1 + 19.4 \left(\frac{d_{pa}}{d_t} \right)^2 \right] \frac{d_{pv}}{d_{pa}}} \tag{S7}$$

The effective axial conductivity is calculated with the correlation (eq. S8) also taken from[5].

$$\frac{\lambda_{ax}}{\lambda_{gas}} = \varepsilon_b + \frac{1 - \varepsilon_b}{0.312\ \varepsilon_b{}^{2.32} + \frac{2}{3}\left(\frac{\lambda_{gas}}{\lambda_s}\right)} + \frac{Re_{d_{pa}} Pr}{2 \left[1 + 19.4 \left(\frac{d_{pa}}{d_t} \right)^2 \right] \frac{d_{pv}}{d_{pa}}} \tag{S8}$$

The wall heat transfer coefficient is calculated by the correlation (eq.S9) taken from[6].

$$h_w = \frac{\lambda_{gas}}{d_{pv}} \left(2\, \varepsilon_b + \frac{1 - \varepsilon_b}{0.0024 \left(\frac{d_t}{d_{pv}} \right)^{1.58} + \frac{1}{3} \left(\frac{\lambda_{gas}}{\lambda_s} \right)} \right) + h_{w,conv}$$

$$Nu = \frac{h_{w,conv} d_{pa}}{\lambda_{gas}} = \begin{cases} Re_{d_{pa}} < 1200\,, & 0.0835 Re_{d_{pa}}^{0.91} \\ Re_{d_{pa}} \geq 1200\,, & 1.23 Re_{d_{pa}}^{0.53} \end{cases} \tag{S9}$$

In all the correlations the particle diameter d_p reported in the equation is calculated as average weighted on the volume of the particle diameters of adsorbent and catalysts pellets.

S1.3 Effective diffusivity in the porous solids

The effective intraporous diffusivity is calculated using correlation taken from [7].

$$D_{eff,i} = \frac{\frac{\varepsilon_p}{\tau}}{\frac{1}{D_{K,i}} + \frac{1}{D_{mix,i}}} \tag{S10}$$

The gas mixture diffusivity used in equation (S10) is calculated as previously reported in equation (S6) while the Knudsen diffusivity (eq. S11) is taken from[7].

$$D_{K,i} = 9700\, r_{pore} \sqrt{\frac{T}{MW_i}} \tag{S11}$$

The ε/τ ratios, porosities and pore radius for methanol and dehydration catalyst are taken from[8,9] and from[10] for the zeolite 3A. These parameters are reported in *Table S1*. In the case of hybrid pellet configuration the parameters are calculated as weighted average on volumetric fraction of methanol and dehydration catalyst.

Table S1. Solid phases parameters.

	Methanol cat.	***Dehydration cat.***	***Adsorbent***	***Unit***
ε_p/τ	0.123	0.152	0.308	(-)
ε_p	0.492	0.607	0.615	(-)
r_{pore}	$9.7\cdot10^{-9}$	$11\cdot10^{-9}$	$1\cdot10^{-6}$	(m)

The intraparticle mass transfer resistance inside the adsorbent particles, in absence of reaction, is accounted by the linear driving force coefficient (K_{LDF}). From a preliminary numerical analysis, it has been found that the macropore diffusion is prevalent and the surface diffusion can be neglected. The correlation used (eq. S12) is taken from[11].

$$\frac{1}{K_{LDF}} = \frac{r_p^2 q_{sat}\rho_{ads}}{15 C_{ads,H_2O} D_{eff,H_2O}} \qquad \text{(S12)}$$

S1.4 Bed void fraction correlation

The Dixon correlations[12] for cylinders and spheres (eq. S13) has been used for the bed void calculation. The resulting void fraction has been calculated as volumetric average between the adsorbent and catalysts particles. The calculated bed void fraction is 0.38.

$$\begin{aligned} \varepsilon_b &= 0.4 + 0.05(d_p/d_t) + 0.412(d_p/d_t)^2 \quad for\ spheres \\ \varepsilon_b &= 0.36 + 0.1(d_p/d_t) + 0.7(d_p/d_t)^2 \quad for\ cylinders \end{aligned} \qquad \text{(S13)}$$

S2. Reaction kinetics constants

Table S2. Kinetic, adsorption and equilibrium constants[13–16].

$K_1 = 2.69 \text{ x } 10^7 \exp\left(\frac{-109900}{RT}\right)$	(mol/(kg$_{cat}$ s bar$^{3/2}$))
$K_2 = 7.31 \text{ x } 10^8 \exp\left(\frac{-123400}{RT}\right)$	(mol/(kg$_{cat}$ s bar))
$K_3 = 4.36 \text{ x } 10^2 \exp\left(\frac{-65200}{RT}\right)$	(mol/(kg$_{cat}$ s bar$^{3/2}$))
$K_4 = 1.0278 \text{ x } 10^{10} \exp\left(\frac{-105000}{RT}\right)$	(mol/(kg$_{cat}$ s)
$K_{CO} = 7.99 \text{ x } 10^{-7} \exp\left(\frac{58100}{RT}\right)$	(bar^{-1})
$K_{CO_2} = 1.02 \text{ x } 10^{-7} \exp\left(\frac{67400}{RT}\right)$	(bar^{-1})
$K_{H_2O/H_2} = 4.13 \text{ x } 10^{-11} \exp\left(\frac{104500}{RT}\right)$	(bar$^{-1/2}$)
$K_{CH_3OH} = 7.9 \text{ x } 10^{-7} \exp\left(\frac{70500}{RT}\right)$	(m^3/mol)
$K_{H_2O} = 0.84 \text{ x } 10^{-4} \exp\left(\frac{41100}{RT}\right)$	(m^3/mol)
$\log K_{eq,1} = \frac{5139}{T} - 12.621$	(bar^{-2})
$\log K_{eq,2} = \frac{-2073}{T} + 2.029$	(-)
$K_{eq,3} = K_{eq,1}K_{eq,2}$	(bar^{-2})
$\ln K_{eq,4} = \frac{4019}{T} + 3.707 \ln T - 2.783 \text{ x } 10^{-3}T + 3.8 \text{ x } 10^{-7}T^2 - \frac{6.561\text{x } 10^4}{T^3} - 26.64$	(-)

S3. Adsorption isotherm

The saturation adsorbent load q_{sat} used[17] in the model is calculated as reported in equation (S18).

$$q_{sat} = q_s \frac{bP_{H_2O}^n}{1 + bP_{H_2O}^n} \quad \text{(S18)}$$

The isotherm parameters (eq. S19) are calculated according to the work of Gabruś et al.[17]. The values of the adsorption constants are reported in *Table S3*.

$$q_s = a_0 + \frac{a_1}{T} + \frac{a_2}{T^2}$$

$$b = exp\left(b_0 + \frac{b_1}{T} + \frac{b_2}{T^2}\right) \quad \text{(S19)}$$

$$n = n_0 + \frac{n_1}{T}$$

Table S3. Adsorption isotherm constants [17].

Constant	***Value***	***Unit***
a_0	0.24486	(mol kg^{-1})
a_1	-29.161	(mol kg^{-1} K)
a_2	743.36	(mol kg^{-1} K^2)
b_0	-10.659	(Pa^{-1} K)
b_1	1969.4	(Pa^{-1} K)
b_2	933.58	(Pa^{-1} K^{-2})
n_0	-0.09294	(-)
n_1	340.56	(K)

References

(1) Pfeffer, R. Heat and Mass Transport in Multiparticle Systems. *Ind. Eng. Chem. Fundam.* **1964**, *3* (4), 380–383.

(2) Wakao, N.; Funazkri, T. Effect of Fluid Dispersion Coefficients on Particle-to-Fluid Mass Transfer Coefficients in Packed Beds. Correlation of Sherwood Numbers. *Chem. Eng. Sci.* **1978**, *33* (10), 1375–1384.

(3) R.C. Reid, J.M. Prausnitz, B. E. P. The Properties of Gases and Liquids, 5th Ed., McGraw-Hill; 2001.

(4) Fuller, E. N.; Ensley, K.; Giddings, J. C. Diffusion of Halogenated Hydrocarbons in Helium. The Effect of Structure on Collision Cross Sections. *J. Phys. Chem.* **1969**, *73* (11), 3679–3685.

(5) Specchia, V.; Sicardi, S. Modified Correlation for the Conductive Contribution of Thermal Conductivity in Packed Bed Reactors. *Chem. Eng. Commun.* **1980**, *6* (1–3), 131–139.

(6) Specchia, V.; Baldi, G.; Sicardi, S. Heat Transfer in Packed Bed Reactors with One Phase Flow. *Chem. Eng. Commun.* **1980**, *4* (1–3), 361–380.

(7) C. H. Bosanquet. British TA Report BR-507, 1944.

(8) Graaf, G. H.; Scholtens, H.; Stamhuis, E. J.; Beenackers, A. A. C. M. Intra-Particle Diffusion Limitations in Low-Pressure Methanol Synthesis. *Chem. Eng. Sci.* **1990**, *45* (4), 773–783.

(9) Berčič, G.; Levec, J. Intrinsic and Global Reaction Rate of Methanol Dehydration over γ-Al_2O_3 Pellets. *Ind. Eng. Chem. Res.* **1992**, *31* (4), 1035–1040.

(10) Gabruś, E.; Witkiewicz, K.; Nastaj, J. Modeling of Regeneration Stage of 3A and 4A Zeolite Molecular Sieves in TSA Process Used for Dewatering of Aliphatic Alcohols. *Chem. Eng. J.* **2018**, *337*, 416–427.

(11) Simo, M.; Sivashanmugam, S.; Brown, C. J.; Hlavacek, V. Adsorption/Desorption of Water and Ethanol on 3A Zeolite in near-Adiabatic Fixed Bed. *Ind. Eng. Chem. Res.* **2009**, *48* (20), 9247–9260.

(12) Dixon, A. G. Correlations for Wall and Particle Shape Effects on Fixed Bed Bulk Voidage. *Can. J. Chem. Eng.* **1988**, *66* (5), 705–708.

(13) G. H. Graaf, P. J. J. Sijtsema, E. J. Stamhuis, G. E. H. J. Chemical Equilibria in Methanol Synthesis. *Chem. Eng. Sci.* **1986**, *41* (11), 2883–2890.

(14) Graaf, G. H.; Stamhuis, E. J.; Beenackers, A. A. C. M. Kinetics of Low-Pressure Methanol Synthesis. *Chem. Eng. Sci.* **1988**, *43* (12), 3185–3195.

(15) Ng, K. L.; Chadwick, D.; Toseland, B. A. Kinetics and Modelling of Dimethyl Ether Synthesis from Synthesis Gas. *Chem. Eng. Sci.* **1999**, *54* (15–16), 3587–3592.

(16) Lu, W. Z.; Teng, L. H.; Xiao, W. De. Simulation and Experiment Study of Dimethyl Ether Synthesis from Syngas in a Fluidized-Bed Reactor. *Chem. Eng. Sci.* **2004**, *59* (22–23), 5455–5464.

(17) Gabruś, E.; Nastaj, J.; Tabero, P.; Aleksandrzak, T. Experimental Studies on 3A and 4A Zeolite Molecular Sieves Regeneration in TSA Process: Aliphatic Alcohols Dewatering-Water Desorption. *Chem. Eng. J.* **2015**, *259*, 232–242.
